# Strain and crystallographic identification of the helically concaved gap surfaces of chiral nanoparticles

**DOI:** 10.1038/s41467-023-39255-1

**Published:** 2023-06-17

**Authors:** Sungwook Choi, Sang Won Im, Ji-Hyeok Huh, Sungwon Kim, Jaeseung Kim, Yae-Chan Lim, Ryeong Myeong Kim, Jeong Hyun Han, Hyeohn Kim, Michael Sprung, Su Yong Lee, Wonsuk Cha, Ross Harder, Seungwoo Lee, Ki Tae Nam, Hyunjung Kim

**Affiliations:** 1 Department of Physics, Sogang University, Seoul, 04107 Korea; 2 Department of Materials Science and Engineering, Seoul National University, Seoul, 08826 Korea; 3 KU-KIST Graduate School of Converging Science & Technology, Korea University, Seoul, 02481 Korea; 4 Deutsches Elektronen-Synchrotron (DESY), Hamburg, 22607 Germany; 5 Pohang Accelerator Laboratory, POSTECH, Pohang, 37673 Korea; 6 Advanced Photon Source, Argonne National Laboratory, Argonne, IL 60439 USA; 7 Department of Integrative Energy Engineering and KU Photonics Center, Korea University, Seoul, 02481 Korea

**Keywords:** Imaging techniques, Nanoparticles

## Abstract

Identifying the three-dimensional (3D) crystal plane and strain-field distributions of nanocrystals is essential for optical, catalytic, and electronic applications. However, it remains a challenge to image concave surfaces of nanoparticles. Here, we develop a methodology for visualizing the 3D information of chiral gold nanoparticles ≈ 200 nm in size with concave gap structures by Bragg coherent X-ray diffraction imaging. The distribution of the high-Miller-index planes constituting the concave chiral gap is precisely determined. The highly strained region adjacent to the chiral gaps is resolved, which was correlated to the 432-symmetric morphology of the nanoparticles and its corresponding plasmonic properties are numerically predicted from the atomically defined structures. This approach can serve as a comprehensive characterization platform for visualizing the 3D crystallographic and strain distributions of nanoparticles with a few hundred nanometers, especially for applications where structural complexity and local heterogeneity are major determinants, as exemplified in plasmonics.

## Abstract (web summary)

Identifying the 3D crystal plane and strain field distributions of nanocrystals with concave surfaces is challenging. Here, the authors analyze the hidden concave gaps of chiral nanoparticles by using coherent Bragg X-ray diffraction imaging.

## Introduction

The three-dimensional (3D) distribution of the exposed surfaces on a nanoparticle (NP) is a determinant of its catalytic, optical, and electronic properties^1–5^. In this regard, the exact indexing of crystallographic planes and determining the corresponding strain distribution inside or on a single NP are important for correlating the properties with the overall morphology and further tuning the stabilized planes by surface doping or ligand coordination^6–10^. However, available methods are limited for revealing the three-dimensional, local arrangement of nanometric features, especially for hidden and concave surfaces. An important but unresolved example is plasmonic NPs, whose light-matter interactions at the subwavelength scale can be tailored by controlling the facets, vertices, and morphology of the metallic NPs. According to recent studies, even an atomic-scale topological protrusion of plasmonic NPs can play a pivotal role in squeezing and focusing a photon into a pico-volumetric space (referred to as pico-cavity)^11,12^. This otherwise impossible light-matter interaction can be further extended to plasmonic chiro-optical properties (e.g., optical chirality)^13,14^, which has implications for advanced applications, such as metamaterials and chiral sensing. An engraving of an atomic-scale 3D chiral morphology on the surface of plasmonic NPs serves as a vista for achieving unnatural optical chirality in the visible regime (i.e., optical helicity density, that is, the projection of the spin angular momentum density on the momentum direction)^1,15^.

Although tomographic imaging with electrons^16,17^ can be used to obtain an atomic resolution, it has limited inspection depth and a trade-off between the resolution and object size. These are the main obstacles in the imaging and crystallographic identification of NPs larger than a few tens of nanometers. In particular, for NPs with complex 3D geometries, the loss of 3D information owing to the limited tilting angle of the sample, which is often referred to as the “missing wedge”^18^, makes it challenging to obtain accurate imaging results and subsequently to use the NPs for quantitative plasmonics.

Since coherent X-rays are utilized from the synchrotron sources and X-ray free electron lasers, 3D coherent X-ray diffraction imaging is available in various geometries^19,20^. 3D imaging in transmission geometry allows in both crystalline and non-crystalline samples, however, it is also constrained by the tilting angle that necessitates prior information about the sample geometry^21^ or the assumption of identical sample copies^22^ for constructing the 3D coherent X-ray diffraction (CXD) patterns. Bragg coherent X-ray diffraction imaging (BCDI)^23,24^ could be a generalized platform for the 3D imaging of NPs with deep concave morphologies and hidden surfaces. Owing to the considerable penetration depth of X-rays and the small angular rotation of the samples required to measure the entire 3D CXD pattern, BCDI is a suitable technique for imaging the complex morphologies of NPs from 100 nm to a few micrometers in size. More importantly, the nature of Bragg diffraction allows BCDI to be used to identify crystallographic and strain information. The defined scattering geometry of the image provides the reference crystallographic orientation^25^ to determine the surface Miller indices and the reconstructed phase in BCDI provides lattice deformation with picometer-scale sensitivity.

As an effort to develop the general method for analyzing complex 3D NPs, we investigated the crystallographic identification of the entire surface and strain-field analysis of a chiral gold NP, whose properties are exotic and have been considered difficult to characterize in terms of atomic surface structures. The investigated NP, which is called 432 helicoid III was a single-crystal with the features of uniformly formed 3D concave chiral gaps with complex geometry and surface Miller indices^1^. We adopted 432 helicoid III as a model because its most complex morphology and chiral plasmonic behavior are not fully understood at the single NP level. The chiral gaps result from the interaction between chiral peptide molecules and the concave high-Miller-index planes exposed during the growth of the NP^26,27^. Although it is of interest to correlate its structure and functional properties, the exact gap structure and the distribution of the surface Miller indices remain unclear, limiting an in-depth understanding of the mechanisms of chiral morphology formation. We found the following important characteristics of the chiral Au NP: (i) the 3D gap surfaces consisted of a complex composition of high-Miller-index planes with a mixed distribution of R and S chirality, (ii) the overall Miller index distribution of the gap surfaces possessed 432-symmetry identical to the NP morphology, and (iii) the strain-field inside the NP was also symmetric, indicating a correlation between the lattice deformation and concave morphology.

## Results

### 3D imaging and crystallographic analysis of concaved gap surface

**Fig. 1:**
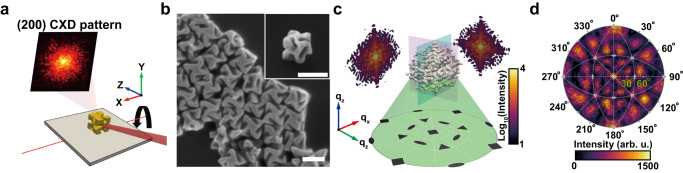
Bragg coherent X-ray diffraction imaging and CXD pattern-based analysis of chiral nanoparticles. **a** Schematic illustration of the BCDI of 432 helicoid III. A focused coherent X-ray was illuminated on 432 helicoid III. The CXD patterns were measured at the Au (200) Bragg reflection with sample rotation along the rocking curve. **b** SEM image of 432 helicoid III NPs showing the cubic outline and chiral gap structures (Scale bar: 200 nm). **c** Measured 3D CXD patterns of 432 helicoid III. **d** Calculated stereographic pole figure projection of the CXD pattern. The {100}, {111}, and {110} points indicated fourfold, threefold, and twofold symmetry, respectively.

We investigated the CXD patterns of 432 helicoid III and then reconstructed them into 3D images. Coherent X-rays were focused on NPs, loaded with the (100) facets facing upward on the substrate (Fig. 1a and Supplementary Fig. 1). The 3D CXD pattern was collected by successive measurements of slices through reciprocal space in the vicinity of the Au (200) Bragg peak by rocking the Bragg angle of the sample. To satisfy the (200) Bragg reflection geometry, a focused X-ray was incident at 19.8° with an X-ray energy of 8.84 keV. Under this condition, the wavevector transfer (**Q**) is parallel to the lattice vectors perpendicular to the (100) facets pointing upward, and the arbitrary rotation of the NP around **Q** is allowed. The measured Au NPs (432 helicoid III) exhibited chiral gaps with concave surfaces positioned at the edges of the cubic morphology, as shown in the scanning electron microscopy (SEM) image in Fig. 1b. Owing to its complex morphology and the directions of the surface, 432 helicoid III showed a 3D CXD pattern with complex interference fringes in multiple directions (Fig. 1c). These directions were mainly distributed in the high-Miller-index region on stereographic projections^28^, in contrast to those of cubic NPs (Fig. 1d and Supplementary Fig. 2)

**Fig. 2:**
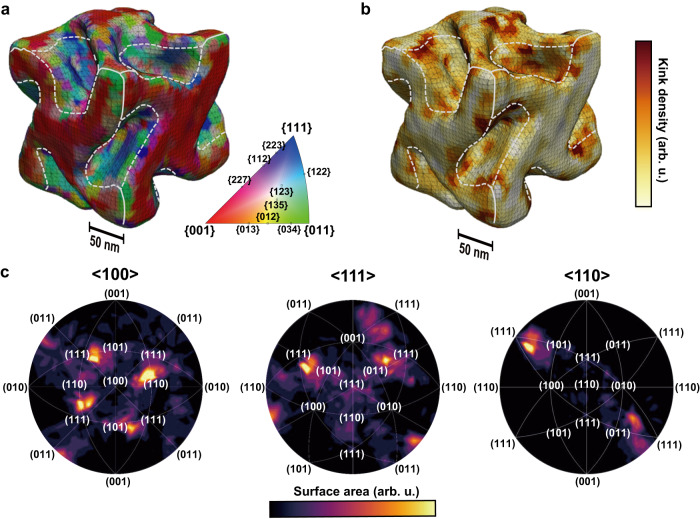
Surface Miller index analysis of 432 helicoid III. **a** Surface Miller indices and **b** kink density of 432 helicoid III. The spatial resolution is 11.6 nm. The white dashed lines indicate the boundaries between the cubic surface (100) and gap regions. The (100), (110), and (111) planes are represented by red, green, and blue, respectively, with the intermediate Miller indices expressed as a color code. The kink density was calculated from the extracted surface Miller indices. **c** Distributions of the surface Miller index on the crystallographic stereographic projection along the <100>, <111>, and <110> directions. The surface area of each orientation of four gaps, three gaps, and one gap is expressed as the brightness on the respective stereographic projections of the Miller indices. Each projection showed approximately fourfold, threefold, and twofold symmetry, matching the point group symmetry of 432 helicoid III.

From the 3D CXD pattern, the Bragg electron density of the NP was reconstructed through an iterative phase-retrieval process^29^. By taking the absolute value of the complex electron density, the 3D morphology of 432 helicoid III was resolved, as shown in Fig. 2a, b. As an example, other types of chiral/achiral NPs are also examined (Supplementary Fig. 3). The crystallographic reference orientations were determined from the **Q** vector and the surface normal direction (**N**) parallel to the lattice vector, which was determined from fitting based on the Terrace-Step-Kink model^30^, which maximizes the terrace ratio of the surface (see Supplementary Fig. 4). The spatial resolution of the imaging result was 11.6 nm, from the full width at half maximum of the point spread function of a 25% iso-surface using the blind deconvolution method^31^ (Supplementary Fig. 5). The size of the pixel of the imaged crystal, denoted as pixel resolution, is estimated at 4.18 nm for our measurement.

For the determination of surface Miller indices considering the resolution of BCDI, we adopted K-means segmentation with root-mean square (RMS) roughness constraints; the upper limit was the spatial resolution to distinguish differences in slopes larger than the resolution, and the lower limit was pixel resolution to prevent sub-pixel artifacts (Supplementary Fig. 6). It provides the results at a given resolution limit without losing geometric features (Supplementary Fig. 7). It is noted that due to the continuously curved characteristic of the gap surface, the actual boundary between different Miller indices is arbitrary by nature. Combined with slight variations at the highly curved NP corner depending on the segmentation starting point, there remains an averaging effect of the atomic fluctuations. However, these variations do not prevent surface identification because surface orientation is determined by combining multiple points (Supplementary Fig. 8). Complementary analysis based on the 3D model, STEM tomography (Supplementary Fig. 9), and scanning helium-ion microscopy^1^ also supports the surface Miller indices analysis.

The imaging result was consistent with SEM images and clearly showed the complex 3D geometry of 432 helicoid III. Two types of regions of the 432 helicoid III surface, flat outer surfaces and concave chiral gaps, were clearly distinguished based on the crystallographic features (Fig. 2a). The flat exterior surfaces mostly consisted of {100}, as expected from the cubic morphology. In contrast, high-Miller-index surfaces were observed on the surface of the concave gaps, in addition to low-Miller-index surfaces such as {110} and {111}. Because the gap has tilted and highly curved surfaces, high-Miller-index surfaces with intermediate slopes are necessary for the continuous surface. The fourfold, threefold, and twofold rotation symmetry axes of 432 helicoid III were also identified, corresponding to [100], [111], and [110], respectively (Supplementary Fig. 10). The chiral gaps were positioned at the [110] edges of the cubic morphology whose surfaces were facing [100]. Each gap had a concave geometry carved toward the inside and tilted inward, determining the overall symmetry of the NP. Each gap can be considered to be a twofold symmetric motif that is located at the center of 12 edges by fourfold and threefold symmetry, combining to form 432-symmetry. Although it is not ideally symmetric, it contrasts with the fact that the geometry of a typical single-crystalline NP follows its lattice symmetry, e.g., a cubic NP has the same $4/{{\mbox{m}}}\overline{3}2/{{\mbox{m}}}$ symmetry as the face-centered cubic crystal lattice (see Supplementary Fig. 11a). This difference originates from the effect of the chiral peptide, which breaks the mirror symmetry of the crystal surfaces. The kink density was calculated from the extracted Miller index and mapped on the 432 helicoid III morphology (Fig. 2b). It was confirmed that chiral kink sites were mainly exposed on the surfaces of the concave gaps. Note that kink density for a cubic NP (Supplementary Fig. 11b) shows kink-free characteristics. From this analysis, we can obtain important evidence for understanding the synthetic mechanism during growth. The lesson is that the chiral gap structure comprises symmetric-preserved high-Miller-index surfaces. For verifying consistency, additional result from another 432 helicoid III is shown in Supplementary Fig. 12.

By converting the surface normal vectors into stereographic projections, the distributions of the surface Miller indices are shown in Fig. 2c. The surface area of each Miller index with color scale is shown for the overall distribution of the Miller indices mapped in two dimensions. The surface Miller indices of four gaps, three gaps, and one gap, observed from <100>, <111>, and <110>, respectively (Supplementary Fig. 10), were plotted on the stereographic projection for each direction. The stereographic projections at <100>, <111>, and <110> exhibit fourfold, threefold, and twofold symmetry, respectively, which matches the 432-point group symmetry of the NP. The lack of threefold symmetry of <111> reflects some imperfection of a chiral gap located in the <111> direction. The chirality of a gap appeared in the Miller index distribution of the regions of the stereographic projections (see, for example, the red region for S chirality in Supplementary Fig. 13a) denoted ${\left(\bar{l}h\bar{k}\right)}^{S}$ and ${\left(h\bar{l}k\right)}^{S}$ (*h* > *k* > *l* > 0), which correspond to the upper and lower parts of the gap faces, respectively. The lack of ${\left(\bar{l}{hk}\right)}^{R}$ and ${\left(h\bar{l}\bar{k}\right)}^{R}$ indicates that the mirror symmetry between the crystal surfaces is broken and that a high-Miller-index with S chirality is preferred for the measured nanocrystal. Strain near the surface and the internal strain distribution of the nanocrystal can provide a fingerprint of the interaction of the chiral molecules, in this case, the peptide, to induce a chiral concave morphology.

### Miller index correlated strain distribution near the chiral gap

**Fig. 3:**
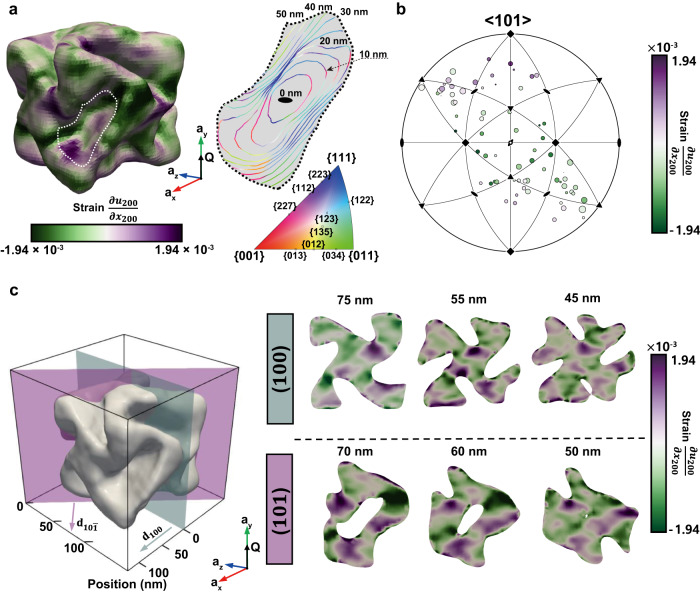
External and internal strain-field distributions induced by geometric symmetry. **a** Surface strain of 432 helicoid III and a detailed contour map of the Miller indices of the chiral gap facing [$10\bar{1}$] (indicated with a white dotted line). **b** Stereographic projection of the surface strain distribution for the [$10\bar{1}$] chiral gap in **a**. The size of each circle indicates the area of the corresponding Miller index. **c** Internal strain-field distribution of 432 helicoid III. The (100) and ($10\bar{1}$) slices of the strain field at different distances from the center are shown. The strain was concentrated in the deep parts of the chiral gap, and its distribution showed symmetry that indicates 432 symmetry in the strain-field.

We analyzed the strain field of 432 helicoid III with regard to the geometry and surface Miller indices. From the lattice displacement field along the **Q** (**a**_**y**_) direction (Supplementary Fig. 14), the strain field was calculated using the **Q** component of the displacement gradient (Fig. 3a). Both tensile and compressive strain were observed on a scale of 10^−3^. In the surface strain distribution of a chiral gap facing <$10\bar{1}$> perpendicular to **Q**, the area indicated with a white dotted line in Fig. 3a, the compressive strain was observed at the concave edge, whereas tensile strain was observed at the side regions. For more detailed information, a contour map of the Miller indices is shown. The Miller index contour map of the gap (marked with a white dotted line) showed that the deepest edge consisted of <00$\bar{1}$>, <100>, and the high-Miller indices connecting <00$\bar{1}$> and <100>, whereas the side regions consisted of high-Miller indices adjacent to {111}. This strain Miller index relationship was confirmed by plotting the strain at each Miller index on a <$10\bar{1}$> stereographic projection (Fig. 3b).

We also discovered that the strain field inside 432 helicoid III exhibited a symmetric pattern directly related to the concave gap structures and symmetry of the morphology. The strain fields of the (100) and ($10\bar{1}$) planes with different depths of 432 helicoid III are shown in Fig. 3c and Supplementary Fig. 15. Compared with the cube (Supplementary Fig. 16), which appeared to be almost strain-free, the strain of 432 helicoid III increased near the chiral gap. Interestingly, the strain field showed twofold symmetry, that is, tensile strain distributed near the chiral gap at the top and bottom and compressive strain at the sides. This indicates that the strain field developed along with the symmetry of 432 helicoid III, because twofold symmetry can only appear when one component of the strain field is considered in fourfold symmetric (100) slices. If there was 432 symmetry in the strain field, we would expect compressive strain in the **a**_**x**_ and **a**_**z**_ directions related to the tensile strain near the top deepest part of the chiral gap in the **a**_**y**_ direction. This result again showed the correlation of the strain field with the surface Miller index, which also indicated a 432-symmetric distribution. Previous studies^32,33^ have reported that adatoms on surface facets induce a strain field in crystals. The building up of a strain field inside 432 helicoid III can result from the steps and kinks as adatoms on the Au surface, which are presumably formed by the interactions of peptides.

### Strain-reflected optical chirality of chiral NP

**Fig. 4:**
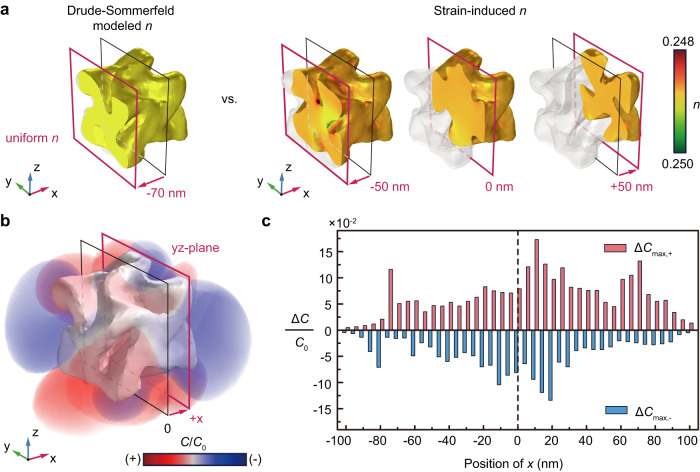
Simulation analysis of the strain and chiroptical characteristics of 432 helicoid III. **a** Three-dimensional spatial color maps of the calculated refractive index based on the Drude-Sommerfeld and strain-induced model at 630 nm. **b** Three-dimensional optical chirality enhancement distributions. **c** Optical chirality difference between the Drude-Sommerfeld and strain-induced models. The dashed vertical line indicates the center position of the cross-section in **b**.

The BCDI results can be directly used to better understand the chiral plasmonic response of a 432 helicoid III. The simulated circular dichroism spectra based on the measured geometry of the 432 helicoid III are consistent with that experimentally measured from colloidal solution, showing a negative peak and a positive peak near the wavelength of 630 nm and 750 nm, respectively (Supplementary Fig. 17). Therefore, we could confirm that the BCDI results represent the morphology of 432 helicoid III responsible for chiroptical properties. Furthermore, we investigated the effect of the strain field, because the mechanical tensile and compressive strain can reconfigure the lattice constant of Au crystals to tune the corresponding dielectric constants of Au^34^. In particular, the intrinsic plasma frequency and core permittivity of Au were found to be adjusted by the atomic lattice constant (see the methods)^35,36^. The spatially variant strains were substituted into the modified Drude-Sommerfeld model and the reconfigured dielectric constants according to the strains were calculated (Supplementary Fig. 18). We then reconstructed 432 helicoid III with the spatially distributed dielectric constants (i.e., the refractive index, $n$), which was compared with a consistently mapped $n$ of 432 helicoid III (Fig. 4a). Given the range of the strain variations, the $n$ of 432 helicoid III can be spatially distributed with a scale of 10^−3^ (see Supplementary Fig. 18 for more details).

This slight change in $n$ negligibly affects the far-field scattering of 432 helicoid III (Supplementary Fig. 19). In particular, the extinction cross-section could be changed by order of magnitude of 10^−16^ owing to the mechanical strain. However, the resultant change in the near-field optical chirality of 432 helicoid III was found to be nontrivial. Over the last decade, the distinct concept of optical chirality (i.e., *C*(**r**) = $-{\varepsilon }_{0}\omega$Im{**E**(**r**) · H^*^(**r**)}/2) has been the gold standard for the nanophotonic-enabled ultrasensitive detection of molecular chirality^13,14,37^. The locally induced plasmonic resonance can strongly enhance and squeeze the optical chirality into a deep-subwavelength-scale (referred to as a chiral hotspot). Consequently, this nanophotonic chiral hotspot in the near-field regime can address the dimensionally mismatched scale between the chiral molecules and the wavelength of interest and thus enormously improve the sensitivity of chiral molecular detection^38,39^. In line with this, the deterministic prediction of the chiral hotspots at the surfaces of plasmonic NPs is crucial because the interactions between the chiral hotspots and molecules are prone to error from the photonic and molecular stochasticity.

At a wavelength of 630 nm, 432 helicoid III exhibited localized surface plasmon resonance (LSPR) (Supplementary Fig. 19b). In this regime, nontrivial corrections were made on the spatial distributions of the near-field enhancement of electric fields by the implementation of a strain-corrected $n$ of a 432 helicoid III NP (Supplementary Fig. 20). As a result, the predictive distributions of optical chirality were corrected accordingly (Fig. 4b). Here, the cross-sectional distributions of optical chirality were taken at each position along the *x*-axis. Note that the spatial position and strength of the optical chirality (chiral hotspots) are heterogeneously distributed along the 3D surface of 432 helicoid III, because they depend on the LSPR mode. We then compared the accessible maximum distributions of optical chirality (*C*_max_ (±)) between a strain-induced $n$ and the constant $n$ of 432 helicoid III ($\triangle$*C*_max_ (±)). We found that $\triangle$*C*_max_ (±) became nonnegligible (on the order of 10^−1^) at a specific position (at approximately +20 nm from the *x*-axis) (Fig. 4c), which could, in turn, leverage the accuracy of the predictive chiral hotspots and the resultant sensitivity of chiral molecular detection by nanophotonics. These chiral hotspots are expected to be further tailored by synthetical control of the chiral morphologies^40^.

## Discussion

Our results demonstrate that BCDI can be used to identify the 3D crystallographic structure of an NP with hidden concave surfaces. By utilizing the feature that BCDI is inherently related to the atomic structure, we successfully determined the surface Miller index and strain-field distributions of 432 helicoid III. An analysis of 432 helicoid III revealed that the chiral gaps consisted of high-Miller-index crystal planes resulting from the enantioselective interactions of peptides and that a strain field correlated to the 432-symmetric morphology existed inside the NP. These results provide not only an understanding of the formation mechanisms but also tuning strategies for chiral morphologies and a blueprint for deep-subwavelength-scale chiral hotspots. Moreover, we anticipate that the BCDI will serve as a precise imaging platform for materials with extended size regimes of tens of nm to a few micrometers, with crystallographic identification and in-situ/operando analysis capability. Real-time imaging of surface orientation during crystal growth^41^ and catalytic reactions^42–44^ can be applied in future research in catalytic, optical, and energy applications. Improvements in the diffraction-limited storage ring source are expected to enable accurate imaging of reconstructed surfaces and microfacets responsible for those physiochemical interactions^45^.

## Methods

### Chemicals

Hexadecyltrimethylammonium bromide (CTAB, 99%), hexadecyltrimethylammonium chloride (CTAC, 98%), L-ascorbic acid (99%), tetrachloroauric(III) trihydrate (HAuCl_4_ · 3H_2_O, 99.9%), L-glutathione (98%), sodium borohydride (NaBH_4_, 99%), and potassium iodide (KI, 99.5%) were purchased from Sigma-Aldrich. High-purity deionized water (18.2 MΩ cm^−1^) was used to prepare aqueous solution.

### Synthesis of 432 helicoid III

432 helicoid III was synthesized as described in ref. ^1^. Spherical seeds (≈2.5 nm) were prepared by mixing 250 μL of 10 mM HAuCl_4_ (99.9%) and 10 mL of 100 mM CTAC (98%), followed by injecting 250 μL of ice-cold NaBH_4_ (99%). The solution was kept at 30 °C for 2 h. The growth solution of octahedral seed was prepared by mixing 250 μL of 10 mM HAuCl_4_, 9.5 mL of 100 mM CTAC, 5 μL of 10 mM KI (99.5%), and 220 μL of 40 mM L-ascorbic acid (99%). 55 μL of a diluted solution of seeds was injected and kept at 30 °C for 15 min. The solution was centrifuged (6708 $\times$*g*, 150 s) twice and redispersed in 1 mM CTAB solution (99%). The growth solution of 432 helicoid III was prepared by mixing 3.95 mL of water, 800 μL of 100 mM CTAB, 100 μL of 10 mM HAuCl_4_, 475 μL of 100 mM l-ascorbic acid, and 5 μL of 5 mM l-glutathione (98%). 50 μL of diluted octahedral seed solution was injected and kept at 30 °C for 2 h. The solution was centrifuged (1,677 $\times$*g*, 60 s) twice and redispersed in 1 mM CTAB solution. Cubic NPs were prepared by the growth of an octahedral seed in a growth solution without l-glutathione.

### Preparation of NP-loaded substrates

Solutions of 432 helicoid III and cubic NPs were concentrated and redispersed in 10 μM CTAB solution. The solutions were spin-coated on a square silicon substrate with a side length of 1.5 cm. The morphology of NPs was measured by scanning electron microscopy (SEM). 100 nm of SiO_2_ protection layer was coated on the substrate by E-beam evaporation.

### BCDI experimental details

BCDI experiments for 432 helicoid III NPs were conducted at P10 beamline, PETRA III (DESY, Germany). Using focused coherent X-ray (1$\times$1.21 μm^2^) by the compound refractive lens with 8.84 keV photon energy, we measured CXD patterns in the vicinity of Au (200) Bragg peak. Each CXD pattern was collected by a 2D detector (Eiger 4 M with 75 μm pixel size) with rotation of NPs on the goniometer along the rocking curve. The sample-to-detector distance (SDD) was 1.84 m. To facilitate high spatial resolution, a wide range (−1.2° to 1.2°) of rocking scans with a step size of 0.01° was measured. BCDIs for cubic gold NPs were measured at 9 C beamline, PLS-II, Korea. The measurement was done with a focused X-ray by KB-mirror (6.8$\times$ 13.1 μm^2^) with 8 keV photon energy. CXD patterns were also measured in Au (200) geometry by 2D detector (Timepix, 55 μm pixel size). The SDD was 0.73 m. The range of the rocking scan was 1.2° with a step size of 0.01°.

In 34-ID-C beamline, APS, USA, a preliminary experiment was conducted. The measurements were done with focused X-ray by KB-mirror (0.45$\times$1.3 μm^2^) with 10 keV photon energy. CXD patterns were also measured in both Au (200) and (111) geometry by a 2D detector (Timepix, 55 μm pixel size). For establishing feasible sample preparation conditions, various sample-to-detector distances and scan ranges were examined.

### Phase-retrieval process

In total, 13 successive scans for the same 432 helicoid III NP were accumulated to improve signal-to-noise ratio. After summing up scans, the diffraction pattern was binned typically (3 by 3) due to the larger than necessary oversampling of the reciprocal space coherent interference patterns caused by the long sample-to-detector distance. Phase-retrieval process^29^ was conducted with combinations of ER and HIO algorithms (20:180) and the total number of iterations was 620. Initial support was square with a random phase. All trials of reconstructions yield similar results. The phase retrieval twin solution of 432 helicoid III were determined by the handedness of chiral morphology. The calculation was accelerated by GPU-based computing (NVIDIA TESLA K20). Phase retrieval for cubic NP was conducted by the same procedure without accumulation in the measurements and binning of the measured data.

### Stereographic projection of CXD pattern

To generate stereographic projection of CXD patterns (Fig. 1d and Supplementary Fig. 2)^28^, the measured pattern was transformed into reciprocal space that conjugated with the basis of sample space. During transformation, the reconstructed phase was treated as zero for the purpose of excluding pattern distortion that originated from lattice strain. For this reason, the transformed CXD pattern became ideally symmetric, and we only consider the upper hemisphere of the pattern. Because there is a trade-off relation between the solid angle of fringe and intensity, radius (*r*) and width of hemisphere (Δ*r*) was selected at near 3rd–4th order fringe to achieve both precise angular information and enough signal-to-noise ratio. For the pattern of 432 helicoid III, *r* = 0.0137 Å^−1^ and Δ*r* = 0.0016 Å^−1^ was selected. For cubic NP, *r* = 0.0203 Å^−1^ and Δ*r* = 0.0016 Å^−1^ were selected. Both stereographic projections were plotted in (100) direction. To present the primary low-Miller -index, the symmetry symbol was overlaid. Indexing of stereographic projection was performed by MTEX toolbox^46^.

### Detailed calculation procedure of coordinate transformation

The transformation of the 3D image is based on the basis transformation of space coordinates^25^. The space coordinate of the 3D image was transformed for a matching basis with direct lattice vectors. The process of the coordinate transformation is shown in Supplementary Fig. 4, which consists of two Rodrigues rotation matrices (**R**_**1**_ and **R**_**2**_). The first step was the calculation of **R**_**1**_, which transforms the basis of lab space basis (I) into **Q**-corrected space basis (II). Rotation matrix **R**_**1**_ that is represented in the figure between (I) and (II) makes wavevector transfer (**Q**) becomes a new **+y′** basis vector of (II). In contrast to the first transformation **R**_**1**_, the calculation of **R**_**2**_ was not based on the actual posture of a NP. To align the surface normal vector (**N**) to one of the direct lattice vectors (**a**_***z***_) by rotating the 3D image around **+y′**, we introduced terrace area (*S*_T_), where the total terrace area of crystal surface in the Terrace-Step-Kink (TSK) model^30^, as a fitting parameter. In the TSK model, the area proportion of each facet with Miller index (*hkl*) is decomposed into micro-facets as below.

1 $$\left({hkl}\right)=u\left(111\right)+v\left(110\right)+w\left(100\right)$$

where Miller index (*hkl*) is sorted from largest to smallest (*h* ≥ *k* ≥ *l* ≥ 0). After decomposition of the surface, terrace area *S*_T_ was calculated for each rotation angle along with **Q** that is parallel to the **+y′**. *S*_T_ is defined as

2 $${S}_{{{\mbox{T}}}}=\sum {A}_{{{\mbox{MF}}}}^{({uvw})}\times \frac{({uvw})}{{{\mbox{|}}}({uvw}){{\mbox{|}}}}\cdot \left(100\right)$$

where ${A}_{{{\mbox{MF}}}}^{({uvw})}$ is an area of decomposed micro-facets. Supplementary Fig. 4b shows the fitting results of the measured *S*_T_. To consider only a flat outer surface, fitting for 432 helicoid III was considered only near the surface segment of the NP presumably considered as (100) surface. The terrace area was calculated within a range of −30° to 30° in 2° steps starting from the arbitral **Q**-normal vector in **Q**-corrected space. The fitting formula was ${S}_{{{\mbox{T}}}}={a}_{1}\sin \left(\omega \theta \right)+{b}_{1}\cos \left(\omega \theta \right)+c$ to fit the area that was changed in the area of two major surfaces perpendicular to each other. **R**_2_ was selected at the maximum point of the *S*_T_ that maximizes the area of the “red” surface, i.e., {100} orientation. To illustrate the procedure for determining the angle of rotation, see Supplementary Movie 1. Final transformed 3D images are shown in (III) in Supplementary Fig. 4 and all direct lattice vectors are parallel to the basis of (III). For this reason, the calculated normal vector of the surface in the sample space is equivalent to the normalized Miller index of the surface. The rotation angle along **Q** was determined by fitting of *S*_T_ where the total terrace area of the crystal surface is in the TSK model^30^.

### Iterative surface segmentation and fitting

Supplementary Fig. 6 shows the schematic flow chart and segmentation results during the early stage of iteration. For every generation, each part was divided into two segments by the K-means segmentation method that minimizes the sum of squared distance within all segments^47^. As shown in Supplementary Fig. 6a, the termination of segmentation was determined by the root-mean-square (RMS) roughness of the segmented surface. Because a surface whose RMS roughness is less than pixel resolution can be considered a perfectly flat surface within a BCDI spatial resolution (11.6 nm), segmentation was terminated if RMS roughness became <1 pixel resolution. Supplementary Fig. 6b–d shows early states of segmentation. Each color represents different segments. After iterative segmentation, total of 1587 flat surfaces were resolved. Each point in surfaces was fitted by the singular value decomposition (SVD) method^48^. Using the SVD method, each segment was fitted to a plane and Miller index was extracted from the normal vector of the fitted plane. Color code for representation of crystallographic orientation use TSL color code in MTEX toolbox that converts orientation into color in HSV color-space. These colors were mapped on the iso-surface of imaged NPs (Fig. 2a, Supplementary Fig. 11a and Supplementary Fig. 12a). All series of image processing were conducted by in-house MATLAB code.

### Calculation of kink density

The kink density of high-Miller-index surface of face-centered-cubic (FCC) gold crystal was evaluated based on TSK model^30^. The atomic surface structure of an ideal crystal facet with Miller index (*hkl*) can be described by the composition of low-Miller-index micro-facets of (111), (100), and (110). Assuming that Miller indices are scaled to the smallest possible integers and *h* ≥ *k* ≥ *l* ≥ 0, Miller index can be decomposed to

3 $$\left({hkl}\right)=l\left(111\right)+\left(k-l\right)\,\left(110\right)+\left(h-k\right)\,\left(100\right)$$

and for the FCC crystal lattice, the number of unit cells is derived as

4 $${n}_{{hkl}}:{n}_{111}:{n}_{110}:{n}_{100}=p:4l:2\left(k-l\right):2\left(h-k\right)$$

where *p* is 2 when *h*, *k*, and *l* are not all odd; *p* is 4 when *h*, *k*, and *l* are all odd. The unit cell area for the Miller index (*hkl*) is

5 $${A}_{{hkl}}=\frac{{a}^{2}}{2}\sqrt{{h}^{2}+{k}^{2}+{l}^{2}}\,{{{{{\rm{for}}}}}}\,{{{{{\rm{h}}}}}},\,{{{{{\rm{k}}}}}},\,{{{{{\rm{l}}}}}}\; {{{{{\rm{not}}}}}} \; {{{{{\rm{all}}}}}}\; {{{{{\rm{odd}}}}}},$$

6 $${A}_{{hkl}}=\frac{{a}^{2}}{4}\sqrt {{h}^{2}+{k}^{2}+{l}^{2}}{{{{{\rm{for}}}}}}\; {{{{{\rm{h}}}}}},\, {{{{{\rm{k}}}}}},\, {{{{{\rm{l}}}}}}\; {{{{{\rm{all}}}}}}\; {{{{{\rm{odd}}}}}},$$

where *a* is the number of kinks is equal to the number of least micro-facet. Therefore, the number of kinks for the area can be derived as

7 $${d}_{{hkl}}^{{{{{{\rm{kink}}}}}}}=\frac{{{\min }}\left\{4l,\, 2\left(k-l\right),\, 2\left(h-k\right)\right\}}{p{A}_{{hkl}}}=\frac{{{\min }}\left\{4l,\, 2\left(k-l \right),\, 2\left(h-k\right)\right\}}{{a}^{2}\sqrt{{h}^{2}+{k}^{2}+{l}^{2}}}$$

The calculated R/S kink density and kink density on the stereographic triangle is illustrated in Supplementary Fig. 13.

### The effect of strain on the dielectric function

We modified Drude-Sommerfeld dielectric function to obtain the dielectric function of the Au crystals, reconfigured by a mechanical strain^34^. Since the lattice of Au atoms can be tuned due to the strain, the dielectric function, which represents the bulk properties of light-matter interaction, should be accordingly changed. This mechanical modification mainly alters the plasma frequency (${\omega }_{{{{{{\rm{p}}}}}}}$) and ${\varepsilon }_{{{{{{\rm{core}}}}}}}$_,_ which are mainly determined by the positive ion cores of the atom. To do this, we began with the modified bulk Drude-Sommerfeld dielectric function that can reflect the effect of nanosized materials^49^. The corresponding Drude-Sommerfeld dielectric function is as follows:

8 $${\varepsilon }_{{{{{{\rm{bulk}}}}}}}\left(\omega \right)={\varepsilon }_{{{{{{\rm{core}}}}}}}\left(\omega \right)-\frac{{\omega }_{{{{{{\rm{p}}}}}}}^{2}}{{\omega }^{2}+{{{{{\rm{i}}}}}}\omega \left({\gamma }_{0}+K\frac{{v}_{{{{{{\rm{F}}}}}}}}{{l}_{{{{{{\rm{eff}}}}}}}}\right)}$$

where ${\varepsilon }_{{{{{{\rm{core}}}}}}}$ corresponds to electric permittivity representing the bound electrons; ${\omega }_{{{{{{\rm{p}}}}}}}$ is plasma frequency; *γ*_0_ indicates the damping constant related to the collision of conduction electrons within the bulk materials. ${\omega }_{{{{{{\rm{p}}}}}}}$ is defined by $\sqrt{n{e}^{2}/({\varepsilon }_{0}{m}_{{{{{{\rm{eff}}}}}}})}$, where $n$, ${m}_{{{{{{\rm{eff}}}}}}},\,e$, and ${\varepsilon }_{0}$ are the electron density, effective mass, elementary charge, and the permittivity of vacuum, respectively.

The main difference between typical and modified Drude-Sommerfeld is the additional term $K{v}_{{{{{{\rm{F}}}}}}}/{l}_{{{{{{\rm{eff}}}}}}}$ in the bracket. This term rationalizes the possibly enhanced collision rate, particularly for the increased ratio of the NP’s surface area to volume. ${v}_{{{{{{\rm{F}}}}}}}$ and ${l}_{{{{{{\rm{eff}}}}}}}$ are the Fermi velocity and the reduced effective mean free path, respectively: ${l}_{{{{{{\rm{eff}}}}}}}$ is defined by $4V/S$, where $V$ is the volume and $S$ is the surface area of the NP. Lastly, $K$ is a dimensionless parameter.

The parameters in Supplementary Table 1 are applied to the modified bulk dielectric function to get the bulk dielectric constants of the nanosized Au crystal^50,51^.

Lastly, we modified ${\omega }_{{{{{{\rm{p}}}}}}}$ to account for the electron density modulation, which is driven by the applied mechanical strain. The modified plasma frequency, ${\omega }_{{{{{{\rm{p}}}}}}}^{{{{{{\rm{strain}}}}}}}$, can be written as^35^

9 $${\omega }_{{{{{{\rm{p}}}}}}}^{{{{{{\rm{strain}}}}}}}=\sqrt{\frac{4{e}^{2}}{{\varepsilon }_{0}{m}_{{{{{{\rm{eff}}}}}}}{a}_{{{{{{\rm{strain}}}}}}}^{3}}}$$

where ${a}_{{{{{{\rm{strain}}}}}}}$ is the deformed lattice constant. In this work, we reconfigured ${a}_{{{{{{\rm{strain}}}}}}}$ according to the measured strain field, shown in Fig. 3b. Furthermore, the plugged mechanical strain affects the dielectric function for the bound electrons ${\varepsilon }_{{{{{{\rm{core}}}}}}}$. Thus, strain-induced ${\varepsilon }_{{{{{{\rm{core}}}}}}}$ can be rewritten as ${\varepsilon }_{{{{{{\rm{core}}}}}}}^{{{{{{\rm{strain}}}}}}}$ and this relation is as follows:^36^

10 $${\varepsilon }_{{{{{{\rm{core}}}}}}}^{{{{{{\rm{strain}}}}}}}\left(\omega \right)=\frac{{\varepsilon }_{{{{{{\rm{core}}}}}}}+2+2\nu \left({\varepsilon }_{{{{{{\rm{core}}}}}}}-1\right)}{{\varepsilon }_{{{{{{\rm{core}}}}}}}+2-\nu \left({\varepsilon }_{{{{{{\rm{core}}}}}}}-1\right)}$$

where $\nu$ is the strain-tuned lattice constant ($\nu={\left({a}_{0}/{a}_{{{{{{\rm{strain}}}}}}}\right)}^{3}$, where ${a}_{0}$ is the lattice constant). Note that we neglected the quantum effects, which can be justified by the relatively large size of the Au NP^52^. According to the following results, the strain-induced dielectric function can be written as

11 $${\varepsilon }_{{{{{{\rm{bulk}}}}}}}^{{{{{{\rm{strain}}}}}}}\left(\omega \right)={\varepsilon }_{{{{{{\rm{core}}}}}}}^{{{{{{\rm{strain}}}}}}}\left(\omega \right)-\frac{{{\omega }_{{{{{{\rm{p}}}}}}}^{{{{{{\rm{strain}}}}}}}}^{2}}{{\omega }^{2}+{{{{{\rm{i}}}}}}\omega \left({\gamma }_{0}+K\frac{{v}_{{{{{{\rm{F}}}}}}}}{{l}_{{{{{{\rm{eff}}}}}}}}\right)}$$

As shown in Supplementary Fig. 18, not only the strain-induced dielectric function (relative permittivity) (Supplementary Fig. 18a) but also the strain-induced complex refractive index was calculated based on the measured strain through the strain-induced dielectric function (Supplementary Fig. 18b).

### Numerical simulation for the strain-induced optical properties

To investigate the effect of strain on both the far-field and near-field optical properties of the Au 432 helicoid III, we carried out the numerical calculation by using a commercial finite-element solver (COMSOL Multiphysics). The simulation domain was encapsulated by the perfectly matched layers (PML). We imported the reconstructed NP model obtained from CDI, and linearly polarized light was illuminated. The dielectric function of Au was embedded by adapting the formula derived from the previous section (i.e., Drude-Sommerfeld and strain-induced model), and the water was used as the host medium with a refractive index of 1.33. The far-field characteristics of 432 helicoid III were estimated through the extinction cross-section (ECS), which is the summation of the scattering cross-section (SCS) and absorption cross-section (ACS). Herein, the SCS was derived by the surface integration of scattered Poynting vector passing the envelope, while ACS was obtained from the integration of the electromagnetic power loss density inside the NP (Supplementary Fig. 19b). The difference in ECS between the Drude-Sommerfeld model and the strain-induced model was also calculated in Supplementary Fig. 19c.

In addition, as shown in Fig. 4 and Supplementary Fig. 20, we analyze the near-field optical properties. The optical chirality $C$ is calculated using the following Eq. (12).

12 $$C\left({{{{{{\bf{r}}}}}}}^{{\prime} }\right)=\frac{{\varepsilon }_{0}\omega }{2}{{{{{\rm{Im}}}}}}\left\{{{{{{{\bf{E}}}}}}}_{{{{{{\bf{sca}}}}}}}\left({{{{{{\bf{r}}}}}}}^{{\prime} }\right)\cdot {{{{{{\bf{H}}}}}}}_{{{{{{\bf{sca}}}}}}}{\left({{{{{{\bf{r}}}}}}}^{{\prime} }\right)}^{*}\right\}$$

where $C\left({{{{{{\bf{r}}}}}}}^{{\prime} }\right)$ of the scattered field at the position ${{{{{\bf{r}}}}}}{{{{{\boldsymbol{=}}}}}}{{{{{{\bf{r}}}}}}}^{{\prime} }$. ${\varepsilon }_{0}$ and $\omega$ correspond to the permittivity at the vacuum and angular frequency of incidence light, respectively. ${{{{{{\bf{E}}}}}}}_{{{{{{\bf{sca}}}}}}}$, and ${{{{{{\bf{H}}}}}}}_{{{{{{\bf{sca}}}}}}}$ indicate scattered electric and magnetic fields, respectively. Both three-dimensional $C$ and ${{{{{{\bf{E}}}}}}}_{{{{{{\bf{sca}}}}}}}$ distributions are displayed through the multiple slice plots calculated from the numerical simulation data (Fig. 4d and Supplementary Fig. 20).

### Reporting summary

Further information on research design is available in the Nature Portfolio Reporting Summary linked to this article.

## Supplementary information

Supplementary Information

Description of Additional Supplementary Files Document

Supplementary Movie. 1.

Reporting Summary

## Data Availability

Raw data were generated at the PETRA III synchrotron radiation facility (P10 beamline) and PLS-II synchrotron radiation facility (9 C beamline). All data supporting the findings of this study are available from the corresponding authors upon request.

## Code availability

All analysis codes are available from the corresponding authors upon request.

## References

[1] Lee, H.-E. et al. Amino-acid- and peptide-directed synthesis of chiral plasmonic gold nanoparticles. *Nature* **556**, 360–365 (2018). 29670265 10.1038/s41586-018-0034-1

[2] Im, S. W. et al. Chiral surface and geometry of metal nanocrystals. *Adv. Mater*. **32**, 1905758 (2020). 10.1002/adma.201905758 31834668

[3] Liz-Marzán, L. M. Tailoring surface plasmons through the morphology and assembly of metal nanoparticles. *Langmuir* **22**, 32–41 (2006). 16378396 10.1021/la0513353

[4] Tian, N., Zhou, Z.-Y., Sun, S.-G., Ding, Y. & Wang, Z. L. Synthesis of tetrahexahedral platinum nanocrystals with high-index facets and high electro-oxidation activity. *Science* **316**, 732–735 (2007). 17478717 10.1126/science.1140484

[5] Grzelczak, M., Pérez-Juste, J., Mulvaney, P. & Liz-Marzán, L. M. Shape control in gold nanoparticle synthesis. *Chem. Soc. Rev.* **37**, 1783–1791 (2008). 18762828 10.1039/b711490g

[6] González-Rubio, G. et al. Micelle-directed chiral seeded growth on anisotropic gold nanocrystals. *Science* **368**, 1472–1477 (2020). 32587018 10.1126/science.aba0980

[7] Huang, L. et al. Shape regulation of high-index facet nanoparticles by dealloying. *Science* **365**, 1159–1163 (2019). 31515391 10.1126/science.aax5843

[8] Ahn, H.-Y. et al. Bioinspired toolkit based on intermolecular encoder toward evolutionary 4D chiral plasmonic materials. *Acc. Chem. Res.* **52**, 2768–2783 (2019). 31536328 10.1021/acs.accounts.9b00264

[9] Johnson, C. L. et al. Effects of elastic anisotropy on strain distributions in decahedral gold nanoparticles. *Nat. Mater.* **7**, 120–124 (2008). 18084296 10.1038/nmat2083

[10] Ma, W. et al. Chiral inorganic nanostructures. *Chem. Rev.* **117**, 8041–8093 (2017). 28426196 10.1021/acs.chemrev.6b00755

[11] Benz, F. et al. Single-molecule optomechanics in “picocavities”. *Science* **354**, 726–729 (2016). 27846600 10.1126/science.aah5243

[12] Baumberg, J. J., Aizpurua, J., Mikkelsen, M. H. & Smith, D. R. Extreme nanophotonics from ultrathin metallic gaps. *Nat. Mater.* **18**, 668–678 (2019). 30936482 10.1038/s41563-019-0290-y

[13] Tang, Y. & Cohen, A. E. Optical chirality and its interaction with matter. *Phys. Rev. Lett.* **104**, 163901 (2010). 20482049 10.1103/PhysRevLett.104.163901

[14] Tang, Y. & Cohen, A. E. Enhanced enantioselectivity in excitation of chiral molecules by superchiral light. *Science* **332**, 333–336 (2011). 21493854 10.1126/science.1202817

[15] Schäferling, M., Dregely, D., Hentschel, M. & Giessen, H. Tailoring enhanced optical chirality: design principles for chiral plasmonic nanostructures. *Phys. Rev. X* **2**, 031010 (2012).

[16] Miao, J., Ercius, P. & Billinge, S. J. L. Atomic electron tomography: 3D structures without crystals. *Science* **353**, aaf2157–aaf2157 (2016). 27708010 10.1126/science.aaf2157

[17] Zhou, J., Yang, Y., Ercius, P. & Miao, J. Atomic electron tomography in three and four dimensions. *MRS Bull.* **45**, 290–297 (2020).

[18] Kováčik, L. et al. A simple Fourier filter for suppression of the missing wedge ray artefacts in single-axis electron tomographic reconstructions. *J. Struct. Biol.* **186**, 141–152 (2014). 24556578 10.1016/j.jsb.2014.02.004 PMC3991334

[19] Miao, J., Ishikawa, T., Robinson, I. K. & Murnane, M. M. Beyond crystallography: diffractive imaging using coherent x-ray light sources. *Science* **348**, 530–535 (2015). 25931551 10.1126/science.aaa1394

[20] Cha, W., Choi, S. & Kim, H. 10.06 - Coherent x-ray diffraction studies of inorganic crystalline nanomaterials. *In*: Comprehensive Inorganic Chemistry III (Third Edition) (Elsevier, 2023).

[21] Xu, R. et al. Single-shot three-dimensional structure determination of nanocrystals with femtosecond X-ray free-electron laser pulses. *Nat. Commun.* **5**, 4061 (2014). 24898682 10.1038/ncomms5061

[22] Ekeberg, T. et al. Three-dimensional reconstruction of the giant mimivirus particle with an X-ray free-electron laser. *Phys. Rev. Lett.* **114**, 098102 (2015). 25793853 10.1103/PhysRevLett.114.098102

[23] Pfeifer, M. A., Williams, G. J., Vartanyants, I. A., Harder, R. & Robinson, I. K. Three-dimensional mapping of a deformation field inside a nanocrystal. *Nature* **442**, 63–66 (2006). 16823449 10.1038/nature04867

[24] Robinson, I. & Harder, R. Coherent X-ray diffraction imaging of strain at the nanoscale. *Nat. Mater.* **8**, 291–298 (2009). 19308088 10.1038/nmat2400

[25] Yang, D., Phillips, N. W. & Hofmann, F. Mapping data between sample and detector conjugated spaces in Bragg coherent diffraction imaging. *J. Synchrotron Radiat.* **26**, 2055–2063 (2019). 31721751 10.1107/S160057751901302X

[26] Kim, H. et al. Glutamylcysteine- and cysteinylglycine-directed growth of chiral gold nanoparticles and their crystallographic analysis. *Angew. Chem. Int. Ed.* **59**, 12976–12983 (2020). 10.1002/anie.202003760 32337812

[27] Shukla, N. & Gellman, A. J. Chiral metal surfaces for enantioselective processes. *Nat. Mater.* **19**, 939–945 (2020). 32747699 10.1038/s41563-020-0734-4

[28] Richard, M.-I. et al. Crystallographic orientation of facets and planar defects in functional nanostructures elucidated by nano-focused coherent diffractive X-ray imaging. *Nanoscale* **10**, 4833–4840 (2018). 29473085 10.1039/c7nr07990g

[29] Fienup, J. R. Phase retrieval algorithms: a comparison. *Appl. Opt.* **21**, 2758 (1982). 20396114 10.1364/AO.21.002758

[30] Van Hove, M. A. & Somorjai, G. A. A new microfacet notation for high-Miller-index surfaces of cubic materials with terrace, step and kink structures. *Surf. Sci.* **92**, 489–518 (1980).

[31] Cherukara, M. J., Cha, W. & Harder, R. J. Anisotropic nano-scale resolution in 3D Bragg coherent diffraction imaging. *Appl. Phys. Lett.* **113**, 203101 (2018).

[32] Kukta, R. V., Kouris, D. & Sieradzki, K. Adatoms and their relation to surface stress. *J. Mech. Phys. Solids* **51**, 1243–1266 (2003).

[33] Muller, P. Elastic effects on surface physics. *Surf. Sci. Rep.* **54**, 157–258 (2004).

[34] Qian, X. & Park, H. S. The influence of mechanical strain on the optical properties of spherical gold nanoparticles. *J. Mech. Phys. Solids* **58**, 330–345 (2010).

[35] Cai, W., Hofmeister, H. & Dubiel, M. Importance of lattice contraction in surface plasmon resonance shift for free and embedded silver particles. *Eur. Phys. J. D.* **13**, 245–253 (2001).

[36] Lermé, J. et al. Influence of lattice contraction on the optical properties and the electron dynamics in silver clusters. *Eur. Phys. J. D.* **17**, 213–220 (2001).

[37] Both, S. et al. Nanophotonic chiral sensing: how does it actually work? *ACS Nano* **16**, 2822–2832 (2022). 35080371 10.1021/acsnano.1c09796

[38] Hendry, E. et al. Ultrasensitive detection and characterization of biomolecules using superchiral fields. *Nat. Nanotech.* **5**, 783–787 (2010). 10.1038/nnano.2010.209 21037572

[39] Zhao, Y. et al. Chirality detection of enantiomers using twisted optical metamaterials. *Nat. Commun.* **8**, 14180 (2017). 28120825 10.1038/ncomms14180 PMC5288493

[40] Cho, N. H. et al. Uniform chiral gap synthesis for high dissymmetry factor in single plasmonic gold nanoparticle. *ACS Nano* **14**, 3595–3602 (2020). 32134639 10.1021/acsnano.9b10094

[41] Yau, A., Cha, W., Kanan, M. W., Stephenson, G. B. & Ulvestad, A. Bragg coherent diffractive imaging of single-grain defect dynamics in polycrystalline films. *Science* **356**, 739–742 (2017). 28522531 10.1126/science.aam6168

[42] Choi, S. et al. In situ strain evolution on Pt nanoparticles during hydrogen peroxide decomposition. *Nano Lett.* **20**, 8541–8548 (2020). 33174748 10.1021/acs.nanolett.0c03005

[43] Kim, D. et al. Active site localization of methane oxidation on Pt nanocrystals. *Nat. Commun.* **9**, 1 (2018). 30143615 10.1038/s41467-018-05464-2 PMC6109038

[44] Kim, Y. Y. et al. Single alloy nanoparticle x-ray imaging during a catalytic reaction. *Sci. Adv.* **7***, eabh0757* (2021). 10.1126/sciadv.abh0757 PMC10938497 34597137

[45] Dietze, S. H. & Shpyrko, O. G. Coherent diffractive imaging: towards achieving atomic resolution. *J. Synchrotron. Rad.* **22**, 1498–1508 (2015). 10.1107/S1600577515017336 26524315

[46] Bachmann, F., Hielscher, R. & Schaeben, H. Texture analysis with MTEX – free and open source software toolbox. *SSP* **160**, 63–68 (2010).

[47] Arun, K. S., Huang, T. S. & Blostein, S. D. Least-squares fitting of two 3-D point sets. *IEEE Trans. Pattern Anal. Mach. Intell.* **9**, 698–700 (1987). 21869429 10.1109/tpami.1987.4767965

[48] Lloyd, S. Least squares quantization in PCM. *IEEE Trans. Inf. Theory* **28**, 129–137 (1982).

[49] Coronado, E. A. & Schatz, G. C. Surface plasmon broadening for arbitrary shape nanoparticles: a geometrical probability approach. *J. Chem. Phys.* **119**, 3926–3934 (2003).

[50] Johnson, P. B. & Christy, R. W. Optical constants of the noble metals. *Phys. Rev. B* **6**, 4370–4379 (1972).

[51] Qiu, L. et al. Observation of plasmon line broadening in single gold nanorods. *Appl. Phys. Lett.* **93**, 153106 (2008).

[52] Liebsch, A. Surface-plasmon dispersion and size dependence of Mie resonance: silver versus simple metals. *Phys. Rev. B* **48**, 11317–11328 (1993). 10.1103/physrevb.48.11317 10007444

