# Supplementary material for: Strain and crystallographic identification of the helically concaved gap surfaces of chiral nanoparticles
Source: Nat Commun. 2023 Jun 17;14:3615. doi: 10.1038/s41467-023-39255-1 (PMC10276881; doi:10.1038/s41467-023-39255-1)
Supplement: Supplementary file 1 — Supplementary Information [file 41467_2023_39255_MOESM1_ESM.pdf]

Supplementary Information

Strain and crystallographic identification of the helically concaved gap surfaces of chiral nanoparticles

Sungwook Choi[1,8], Sang Won Im[2,8], Ji-Hyeok Huh[3], Sungwon Kim[1], Jaeseung Kim[1], Yae-Chan Lim[2], Ryeong Myeong Kim[2], Jeong Hyun Han[2], Hyeohn Kim[2], Michael Sprung[4], Su Yong Lee[5], Wonsuk Cha[6], Ross Harder[6], Seungwoo Lee[3,7], Ki Tae Nam[2]*, Hyunjung Kim[1]*

[1]Department of Physics, Sogang University, Seoul 04107, Korea.

[2]Department of Materials Science and Engineering, Seoul National University, Seoul 08826, Korea

[3]KU-KIST Graduate School of Converging Science & Technology, Korea University, Seoul 02481, Korea

[4]Deutsches Elektronen-Synchrotron (DESY), Hamburg 22607, Germany

[5]Pohang Accelerator Laboratory, Pohang 37673, Korea

[6]Advanced Photon Source, Argonne National Laboratory, Argonne, Illinois 60439, USA

[7]Department of Integrative Energy Engineering and KU Photonics Center, Korea University, Seoul 02481, Korea

[8]These authors contributed equally: Sungwook Choi, Sang Won Im.

*e-mail: nkitae@snu.ac.kr, hkim@sogang.ac.kr

This PDF file includes:

Supplementary Text

Supplementary Figures 1 to 20

Supplementary Table 1

Other Supplementary Information for this manuscript includes the following:

Supplementary Movie 1

Supplementary Text

Consistency of the strain-field distribution

We verified that another 432 helicoid III NP showed a symmetric strain-field distribution consistent with the main results (Fig. 3c, Supplementary Fig. 12, and Supplementary Fig. 15). There are tensile strain distributions near the chiral gap at the top and bottom and compressive strain at the sides. Another NP was measured under the same conditions, except the rocking range (-0.6°- 0.6°).

Supplementary Fig. 1.

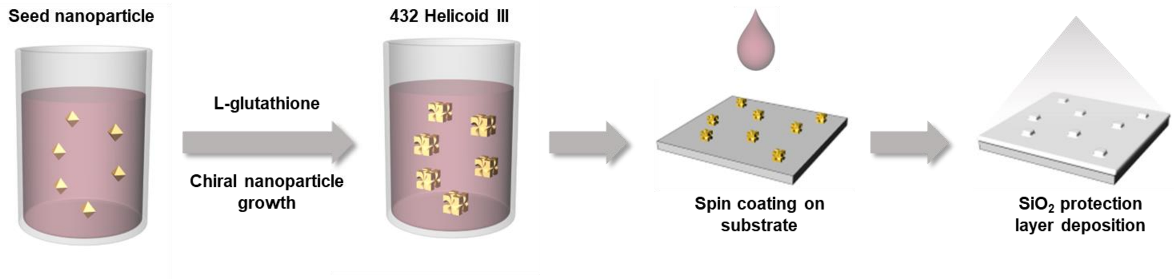

Supplementary Fig. 1 | Preparation of 432 helicoid III nanoparticles. Schematic description of 432 helicoid III nanoparticle sample preparation.

Supplementary Fig. 2.

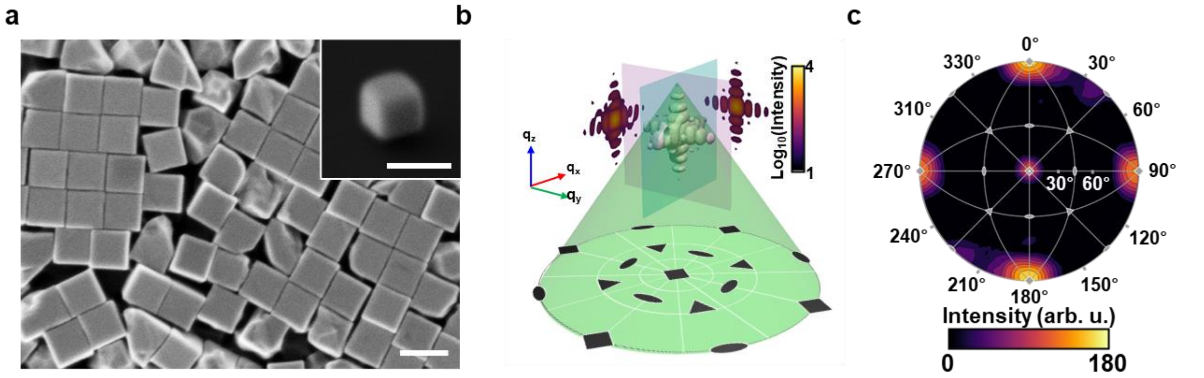

Supplementary Fig. 2 | Scanning electron microscopy (SEM) image and stereographic pole figure projection analysis for cubic NP. a, Scanning electron microscopy (SEM) image of cubic nanoparticles (Scale bar: 200 nm). **b,** Measured three-dimensional CXD patterns cubic. The stereographic pole figure projection (green) is calculated for the upper slice of the hemisphere. **c,** Stereographic pole figure projection of the CXD pattern of the cube. The {100}, {111}, and {110} points are indicated with fourfold, threefold, and twofold symmetry, respectively.

Supplementary Fig. 3.

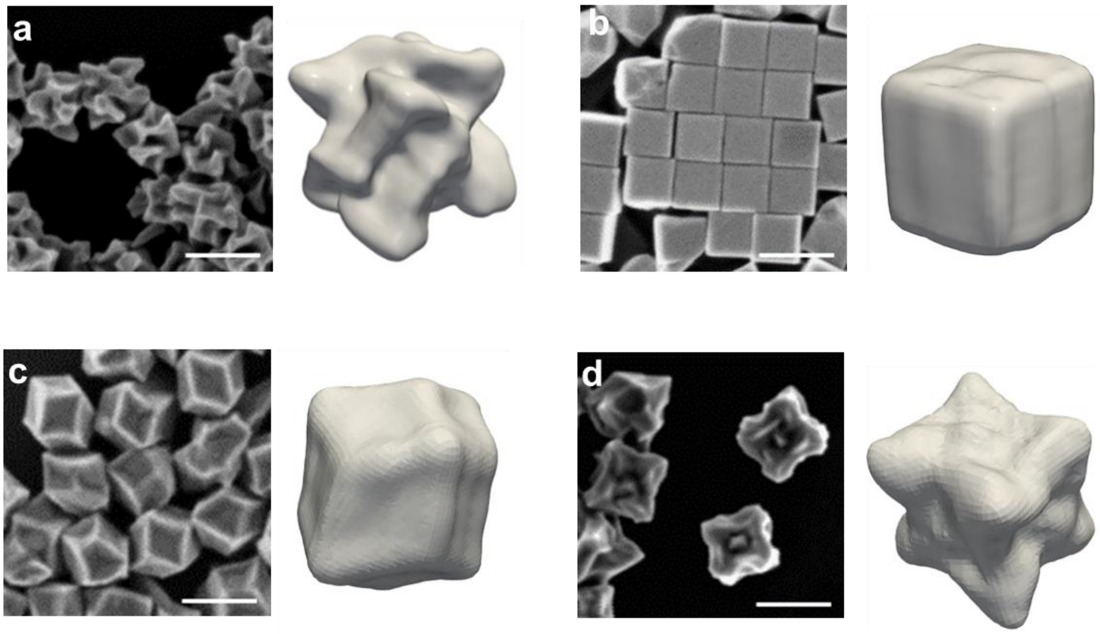

Supplementary Fig. 3 | Scanning electron microscopy (SEM) image and imaging results for various chiral/achiral nanoparticles. a-d, SEM images and corresponding 3D imaging results for (**a**) 432 helicoid III with early growth stage, (**b**) achiral cube, (**c**) the achiral concave rhombic dodecahedron and (**d**) 432 helicoid I (all scale bar: 200 nm).

Supplementary Fig. 4.

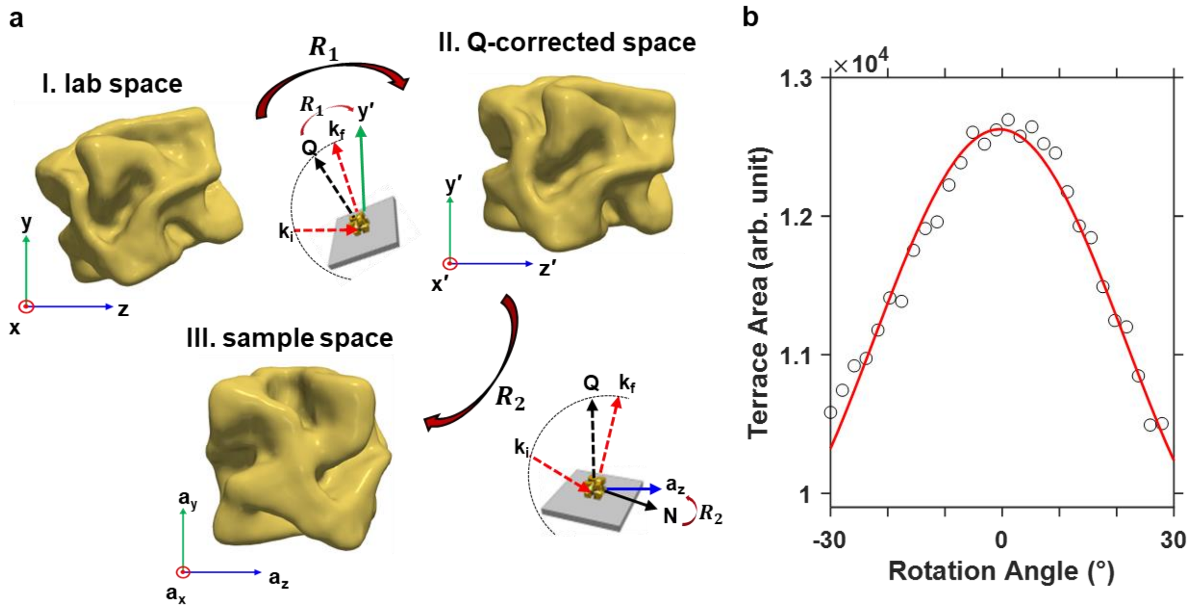

Supplementary Fig. 4 | Coordinate transformation process of 432 helicoid III. a, The coordinate transformation process from the lab space to the sample space for 432 helicoid III. 3D images in lab space (I) are transformed into images in sample space (III) using two Rodrigues' rotation matrices R_1 (I to II) and R_2 (II to III). The figures between (I-III) show the relation between basis and other geometrical indicators. **b,** Fitting of the terrace area S_T for calculation of transformation matrix R_2 for 432 helicoid III.

Supplementary Fig. 5.

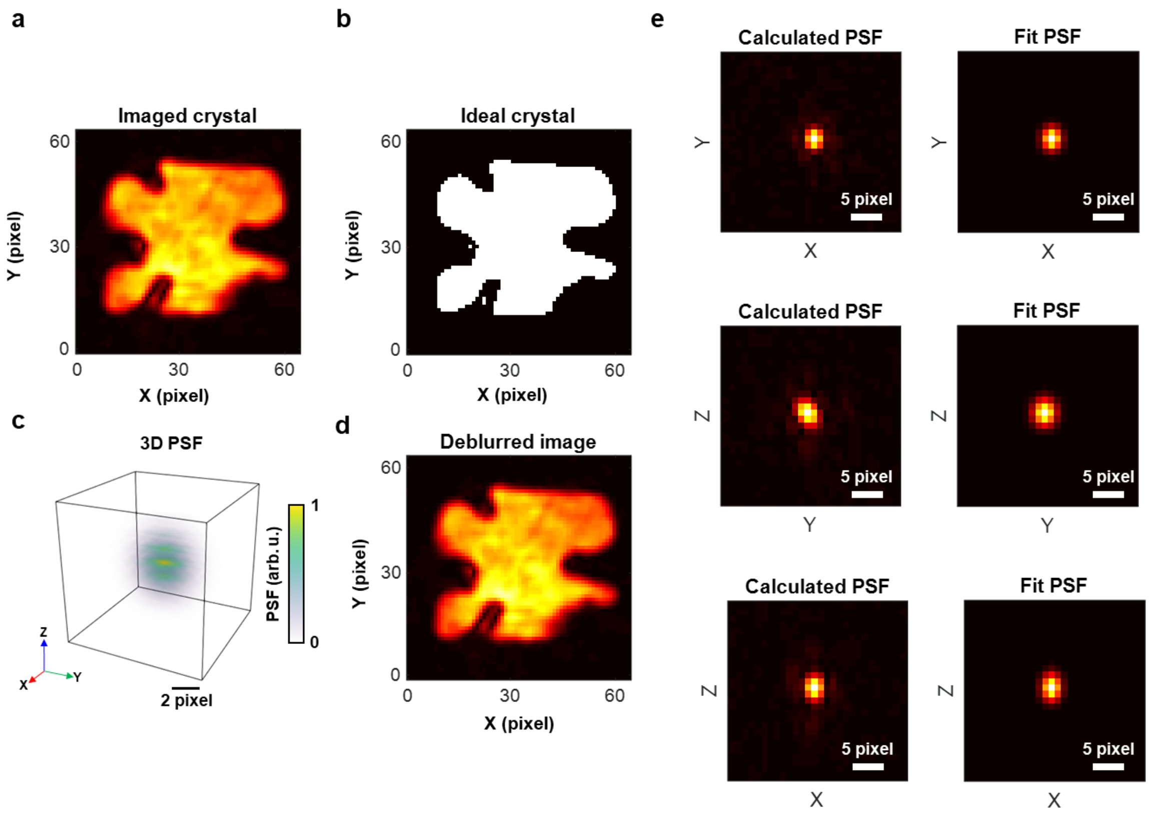

Supplementary Fig. 5 | Calculation of the spatial resolution using the point spread function. **a-d,** Calculation result of the 3D point spread function by blind deconvolution method. XY-center slice of images and the 3D PSF function are shown. Convolution between (**b**) virtual ideal crystal and (**c**) PSF function derives (**d**) deblurred image identical with (**a**) imaging results. **e,** Fitting result of the 3D PSF function. Full-width-half-maximum (FWHM) of the Gaussian represents the spatial resolution for the corresponding direction.

Supplementary Fig. 6.

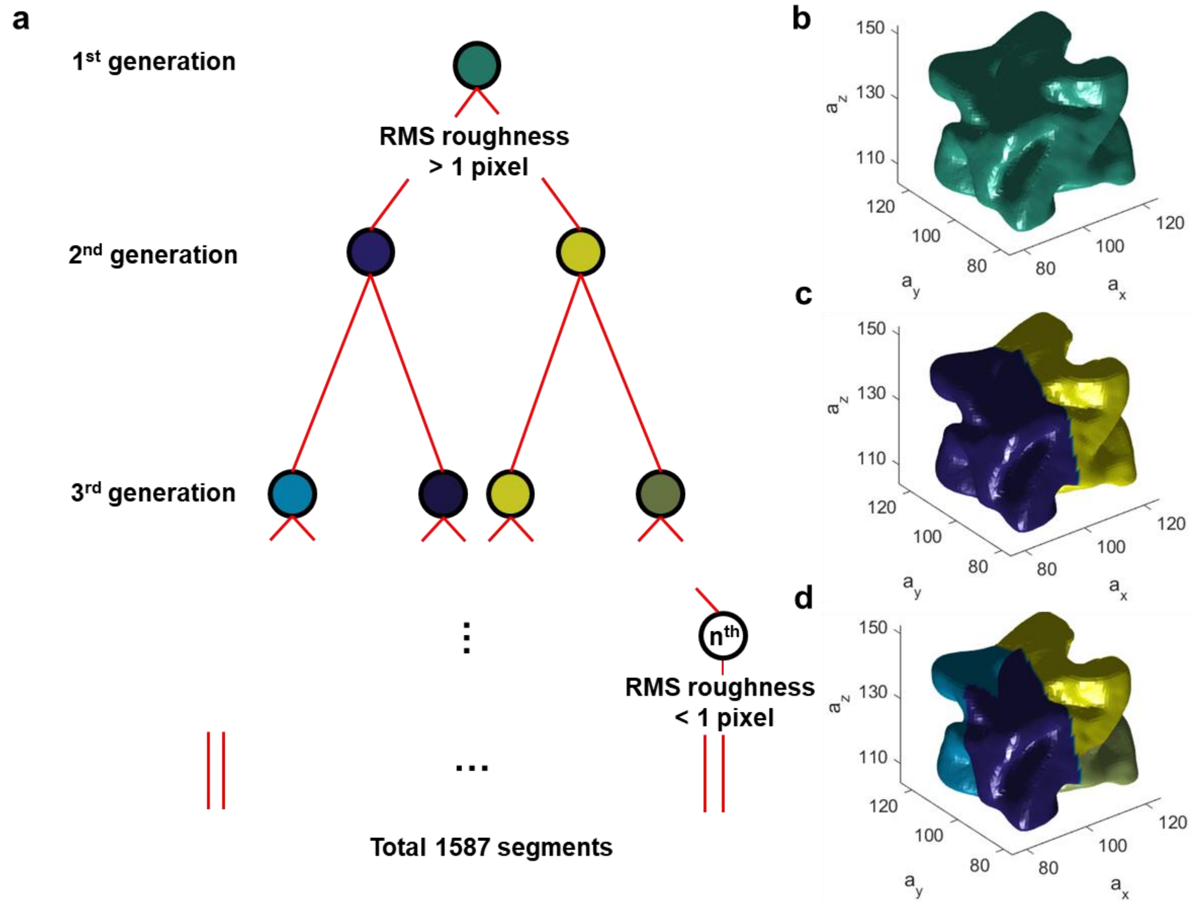

Supplementary Fig. 6 | Iterative K-means surface segmentation of 432 Helicoid III nanoparticle. a, Schematic flow chart of the iterative segmentation algorithm. Each segment is divided into two sub-segments by the K-means clustering method unless root-mean-square roughness becomes less than 1 pixel (4.175 nm). **b-d,** First three generations of the iterative surface segmentation algorithm. Each subsegment is represented with a different color.

Supplementary Fig. 7.

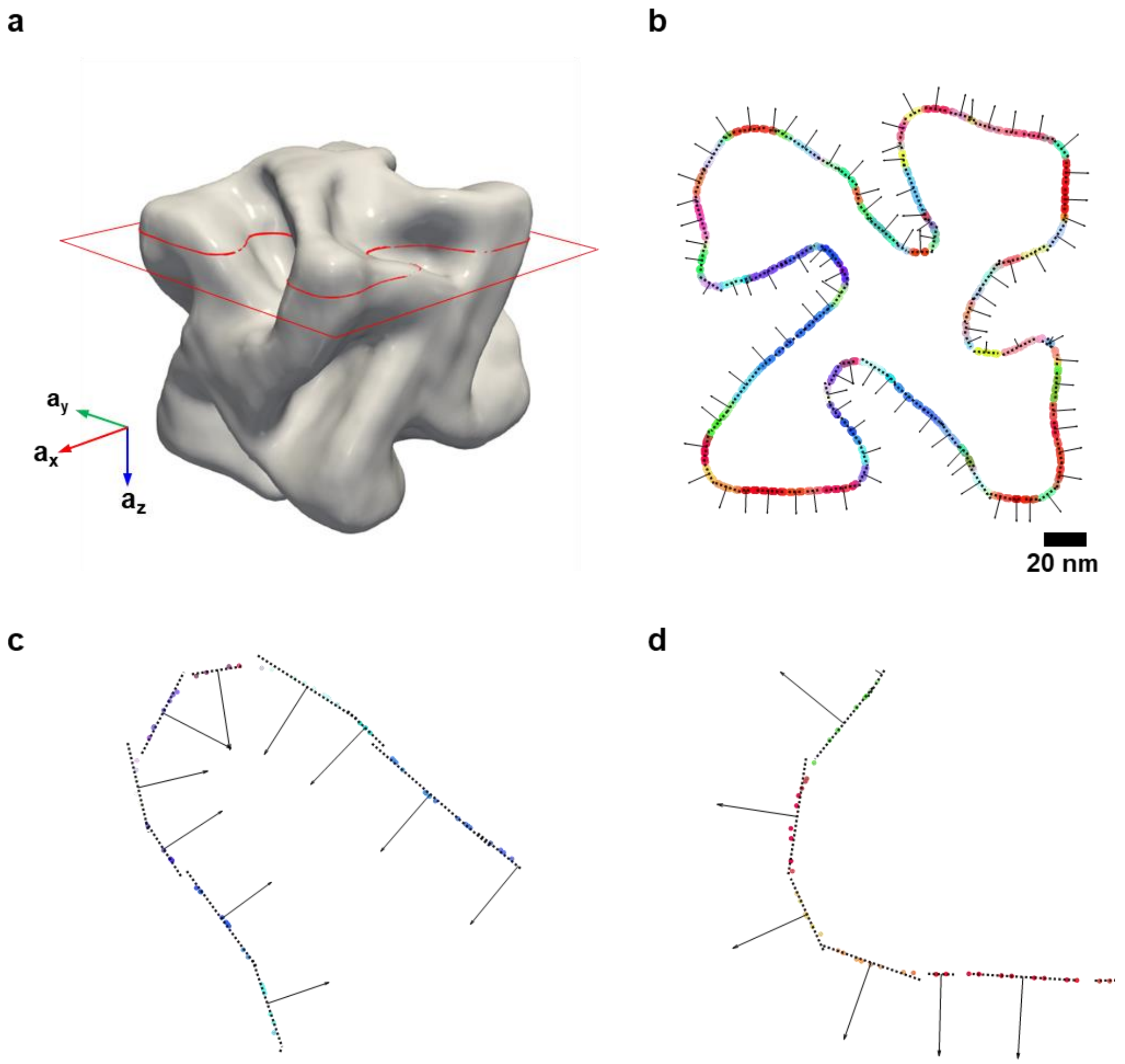

Supplementary Fig. 7 | Surface segmentation result. a, The imaging result and a slice of -55 nm from the center along the z-direction. **b**, the projected result of surface segmentation. Normal vectors are projected onto the xy-plane. **c,** Enlarged projected segmentation result for the chiral gap, **d,** the corner between the flat outer surface and chiral gap.

Supplementary Fig. 8

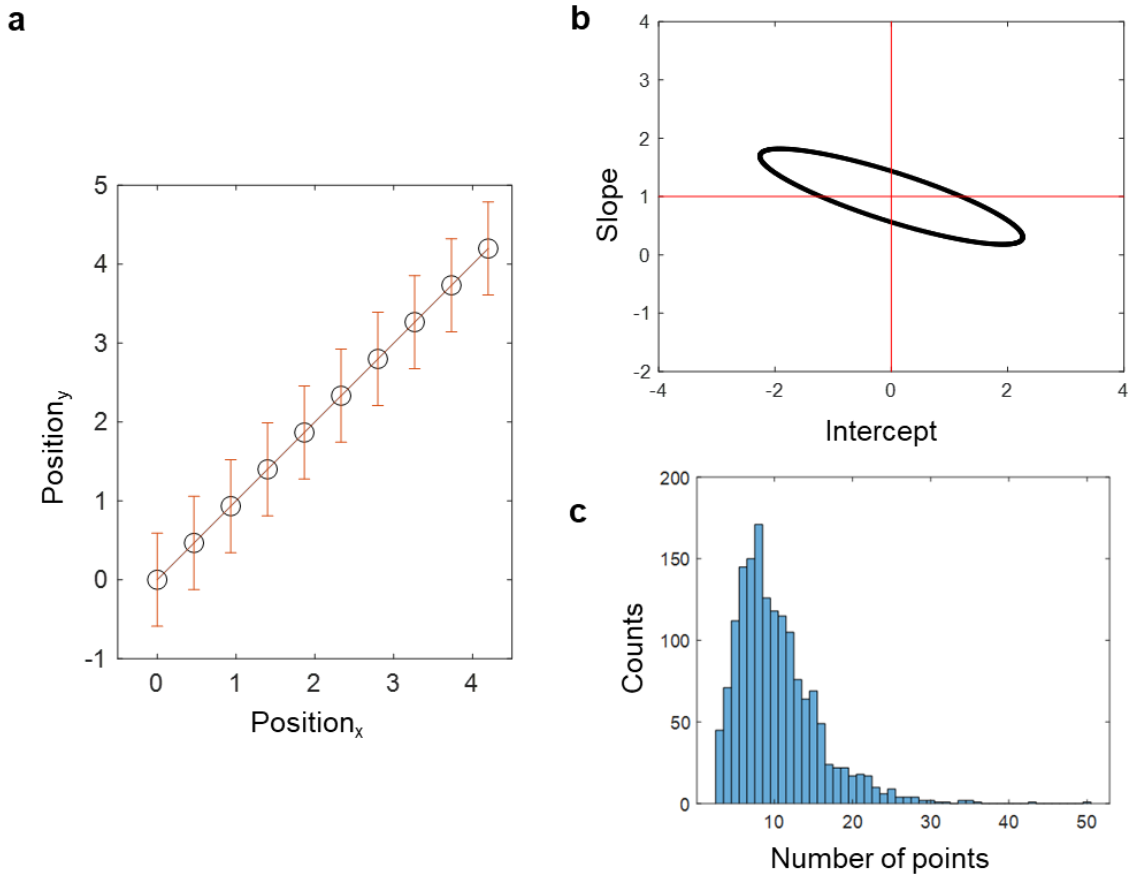

Supplementary Fig. 8 | Calculating confidence ellipse for simplified dimension. a, distribution of 10 points on a line. The ratio between nearest-distance and spatial resolution error is equivalent to the measured data. **b,** Calculated confidence ellipse for 10 points (**a**). The variation of the slope can be specified within ~10 deg from the original estimation. Note that a simplified two-dimension with a smaller degree of freedom drives larger angular uncertainty than a real three-dimensional case. **c,** Histogram representing the population of segments along consisting number of points.

Supplementary Fig. 9

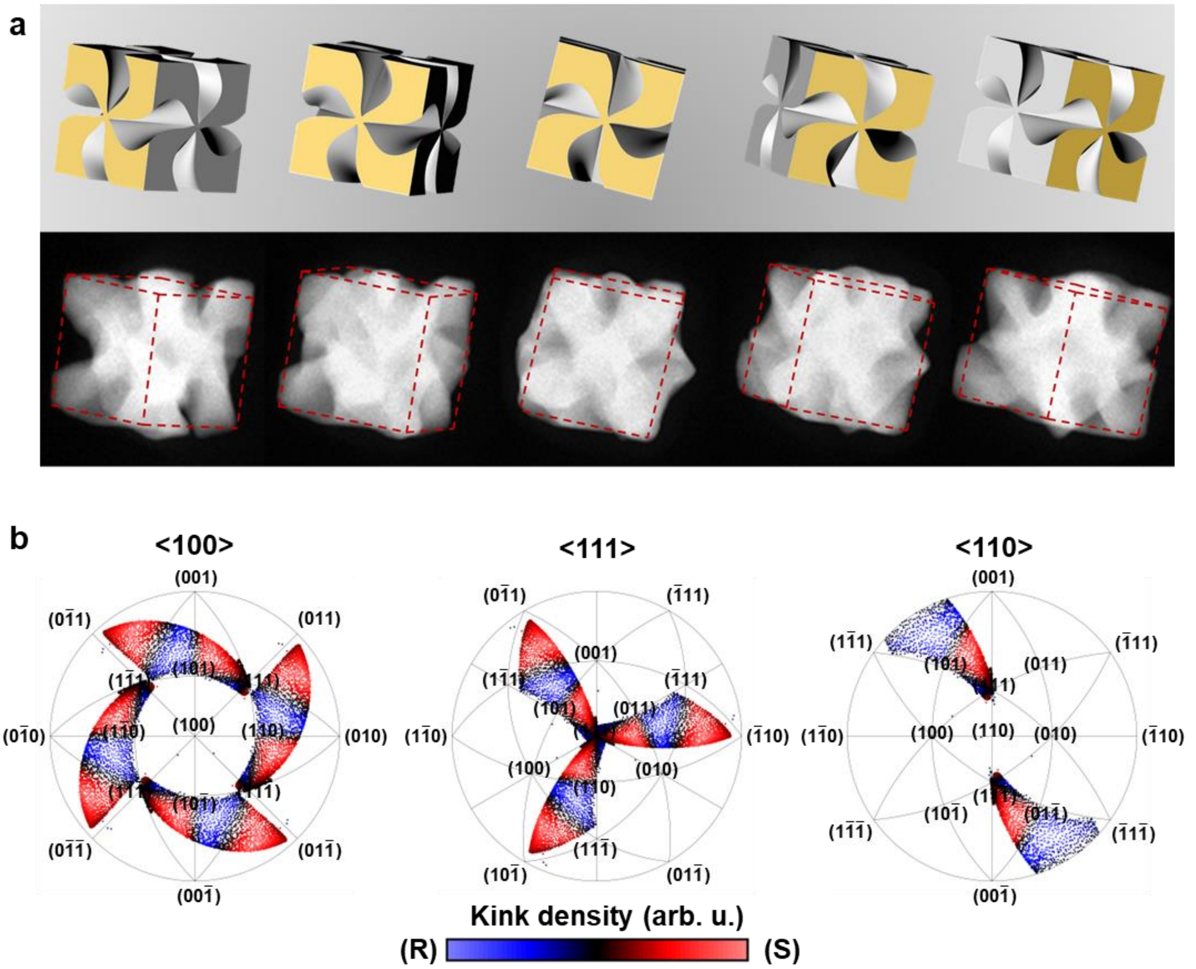

Supplementary Fig. 9 | Complementary three-dimensional analysis for surface Miller-indices analysis a, HADDF-STEM tomography of 432 helicoid III. Tilt series of HAADF-STEM for 432 helicoid III are shown. The orientations of the nanoparticle are indicated with 3D models and the cubic outline is indicated with red dotted lines **b,** Surface orientation is extracted from four, three, and one gap to analyze the surface Miller index and kink density. Each maps are projected along the <100>, <111>, and <110> directions respectively. Brightness indicates the density of kink sites. The s-chiral plane is indicated with red and the R-chiral plane with blue.

Supplementary Fig. 10.

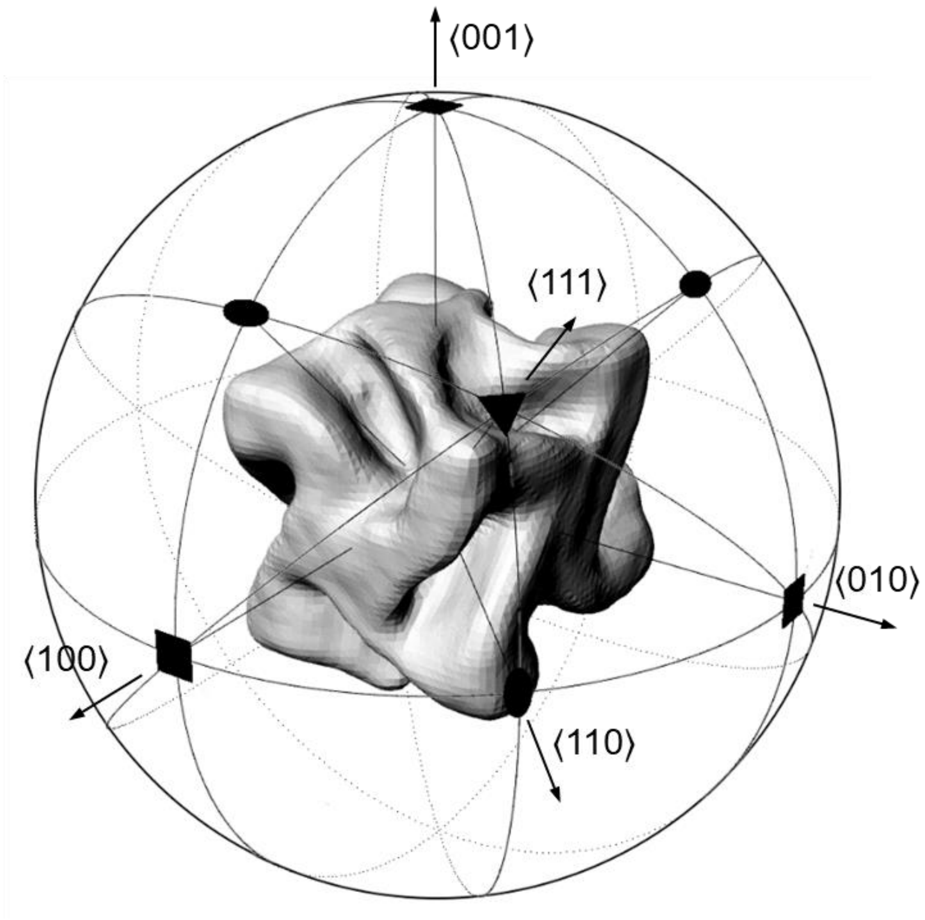

Supplementary Fig. 10 | Reconstructed 3D geometry of 432 helicoid III nanoparticles. Symmetry symbols on the sphere indicate fourfold, threefold, and twofold symmetry axes of 432 point group symmetry.

Supplementary Fig. 11.

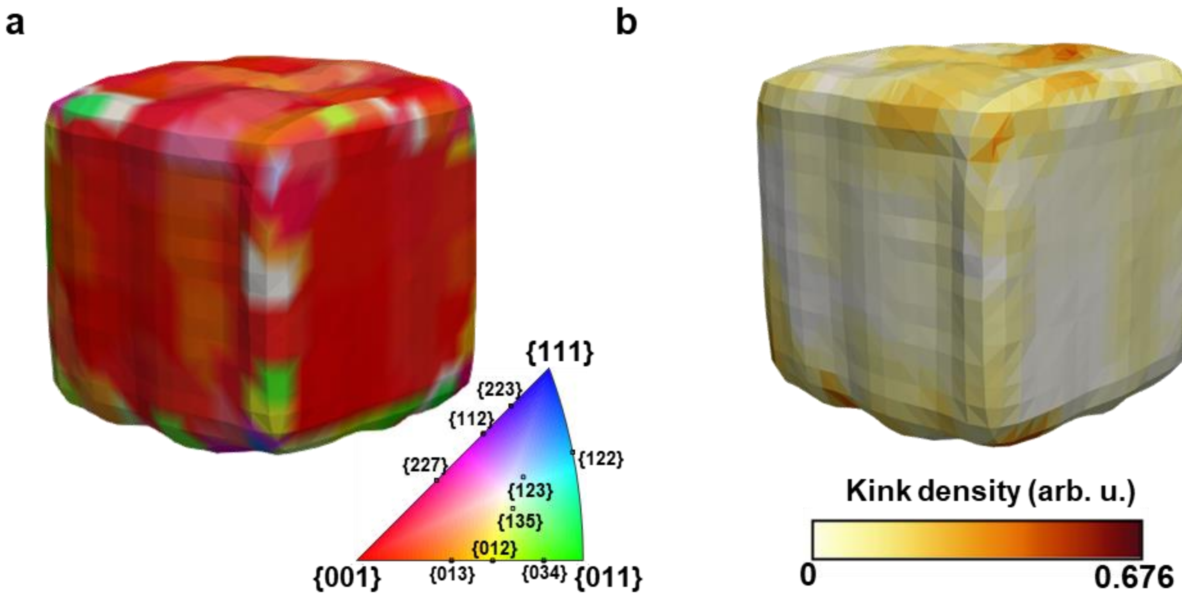

Supplementary Fig. 11 | Crystallographic identification for cubic nanoparticle. a, Surface Miller-index of cubic gold nanoparticle. **b,** Surface kink density of cubic nanoparticle.

Supplementary Fig. 12.

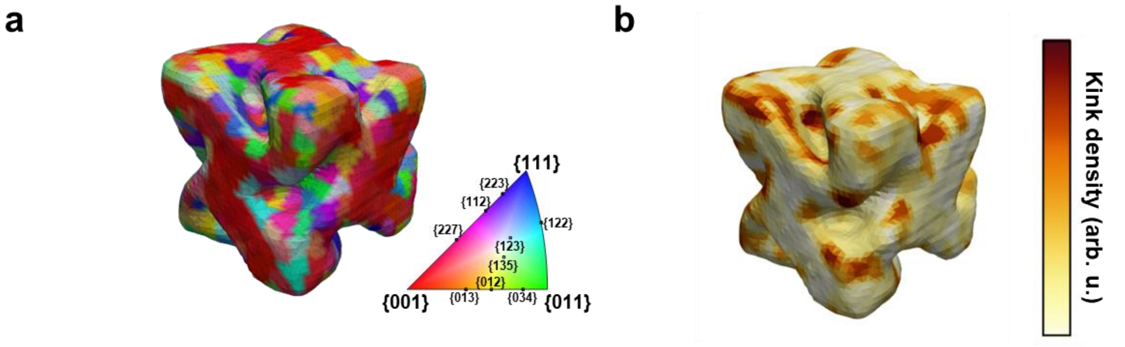

Supplementary Fig. 12 | Surface Miller-index analysis for another 432 helicoid III nanoparticle. a, Surface Miller indices and **b**, kink density express on the imaging result of another 432 helicoid III.

Supplementary Fig. 13.

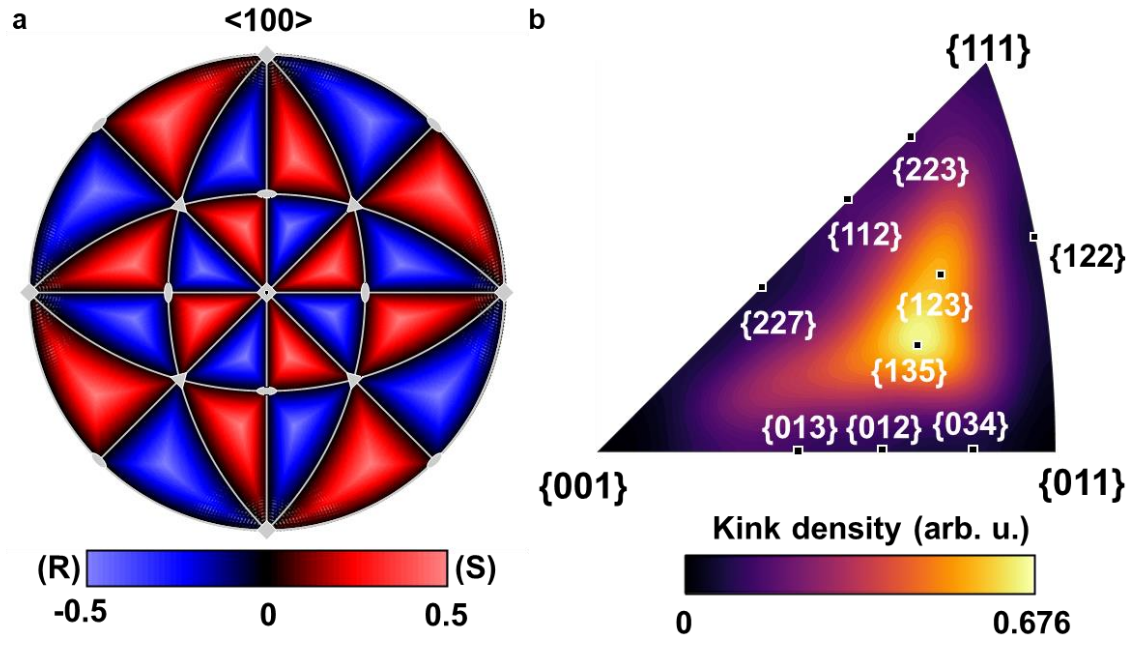

Supplementary Fig. 13 | Stereographic description of R/S chirality and kink density. a, Stereographic projection of R/S kink density with symmetry symbols**. b,** Kink density on the stereographic triangle.

Supplementary Fig. 14.

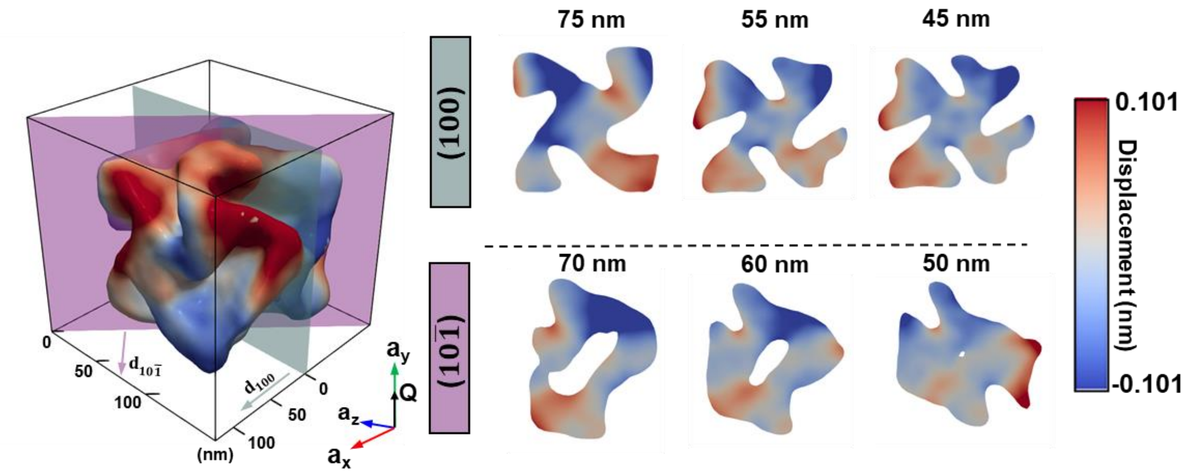

Supplementary Fig. 14 | Displacement field of 432 helicoid III. Lattice displacement-field slices of the 432 helicoid III for (100) and (10$\bar{1}$) planes. Each distance from the center is indicated at the outline of the nanoparticle and slices. (Same slices in Fig. 4a).

Supplementary Fig. 15.

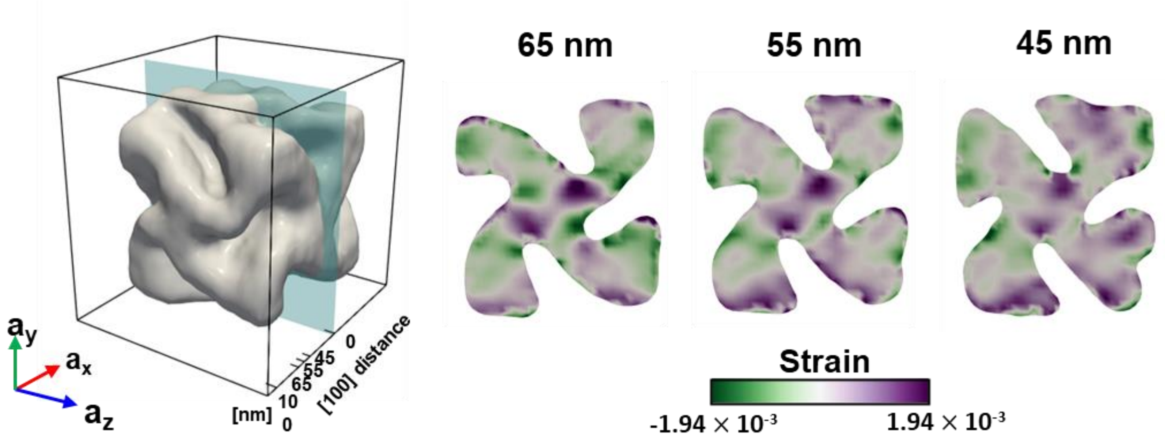

Supplementary Fig. 15 | Strain analysis of another 432 helicoid III. Strain slices of the typical chiral gap with distance (**a_x**) from the center. The same symmetric strain distribution near the chiral gap is observed.

Supplementary Fig. 16.

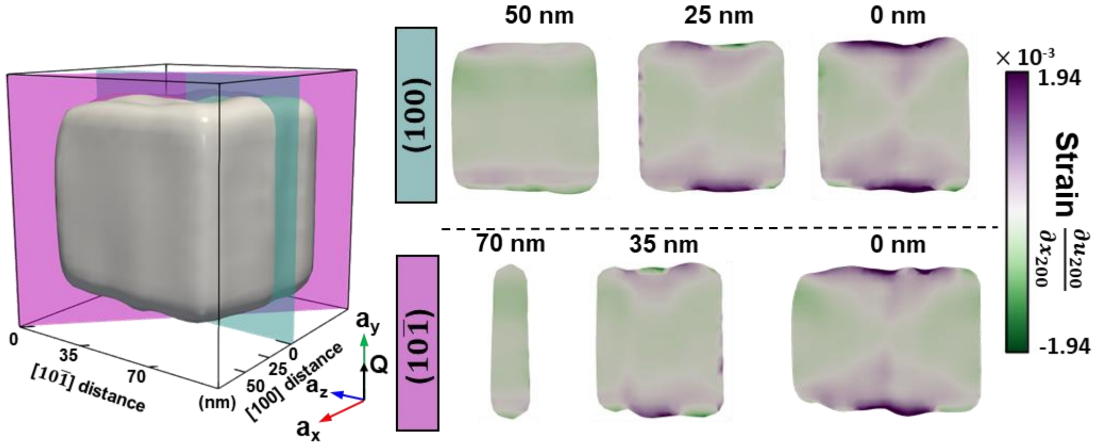

Supplementary Fig. 16 | Strain slices of cubic NP. Strain slices of the cube NP from the center for (100) and (10$\bar{1}$) planes. Each distance is indicated at the outline of the nanoparticle and slices.

Supplementary Fig. 17.

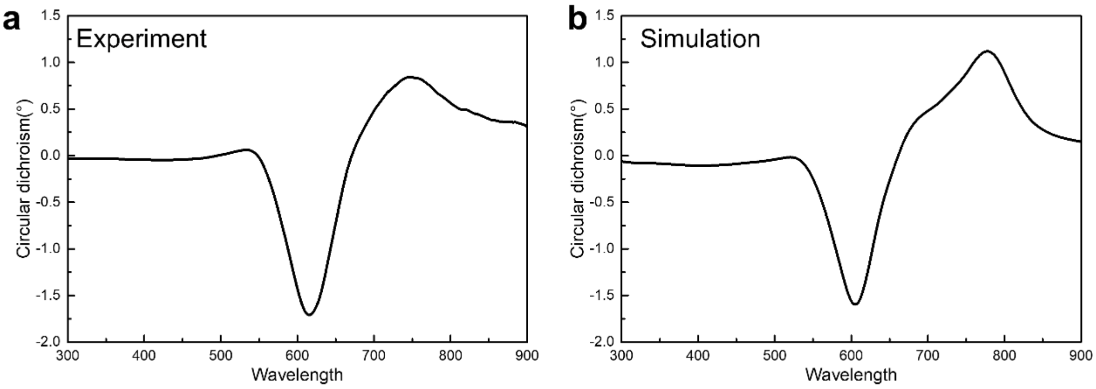

Supplementary Fig. 17 | Circular dichroism spectra of 432 helicoid III. a, Measured circular dichroism spectra of aqueous colloidal solution of 432 helicoid III. **b,** Calculated circular dichroism and extinction spectra based on BCDI result and using finite element solver. The medium surrounding nanoparticle was assumed to be water.

Supplementary Fig. 18.

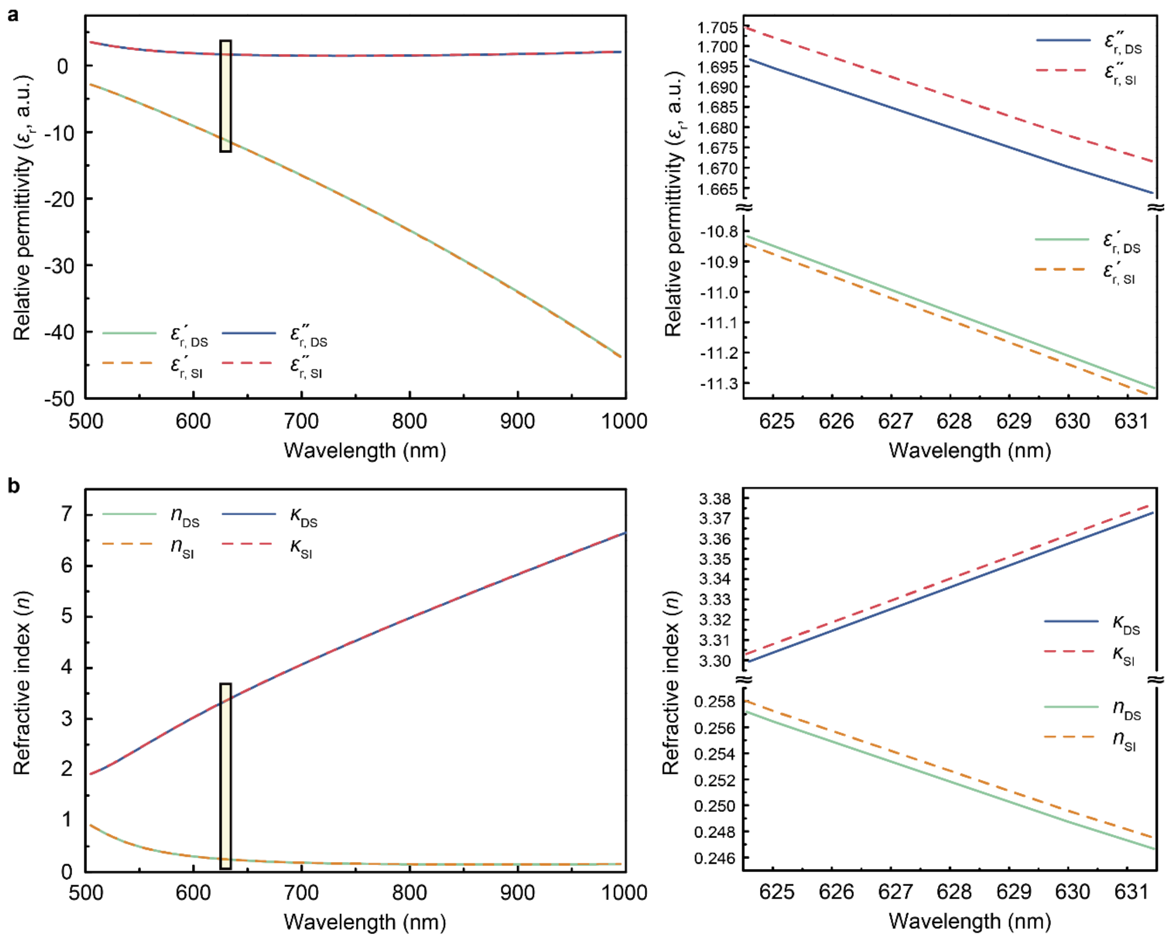

Supplementary Fig. 18 | Comparison of gold dispersion between the typical Drude-Sommerfeld and strain-induced modified model. a, Comparison of dielectric function **b,** Comparison of complex refractive index. The range marked in a box (625-631 nm) is expanded on the right.

Supplementary Fig. 19.

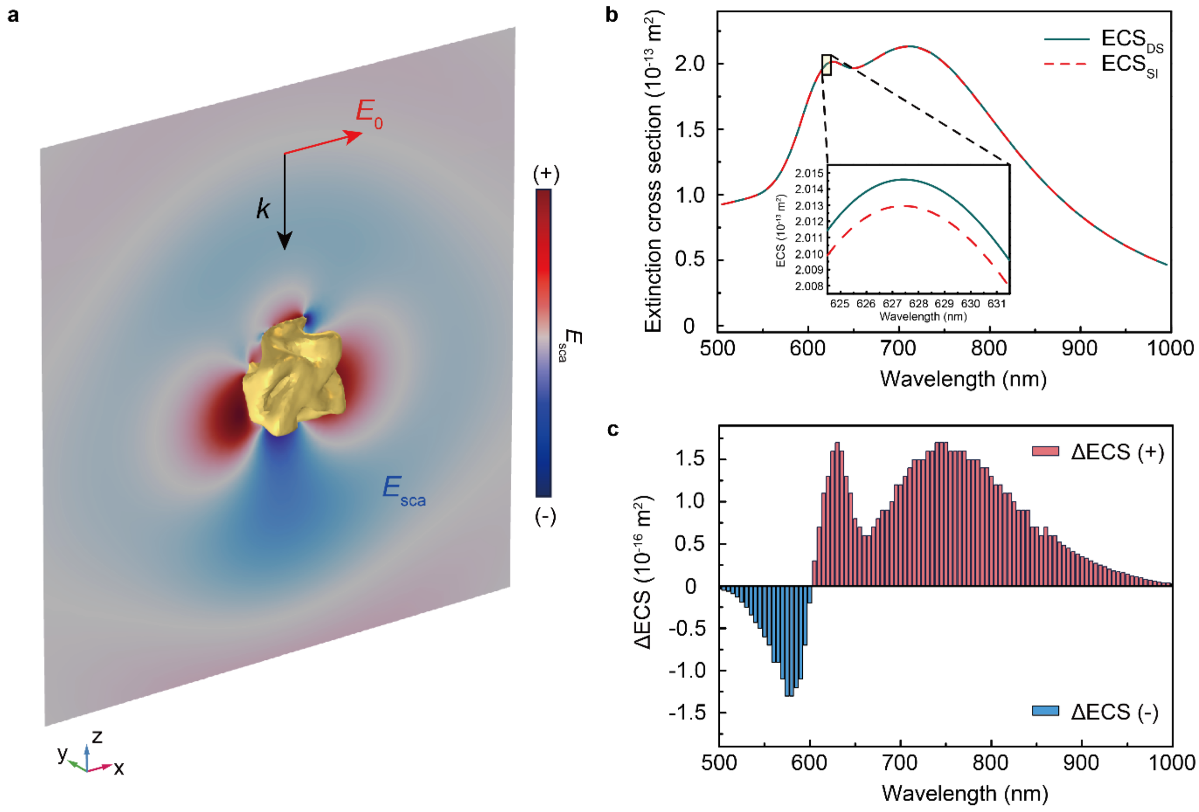

Supplementary Fig. 19 | Simulated far-field extinction cross-section of 432 helicoid III nanoparticle based on BCDI result. a, The scattered electric field of 432 helicoid III under the incidence of x-axis linearly polarized light at 630 nm **b,** Comparison of extinction cross-section of 432 helicoid III between the Drude-Sommerfeld (ECS_{DS}) and strain-induced model (ECS_{SI}) **c,** and the difference between two models (ECS_{DS} -ECS_{SI} =ΔECS).

Supplementary Fig. 20.

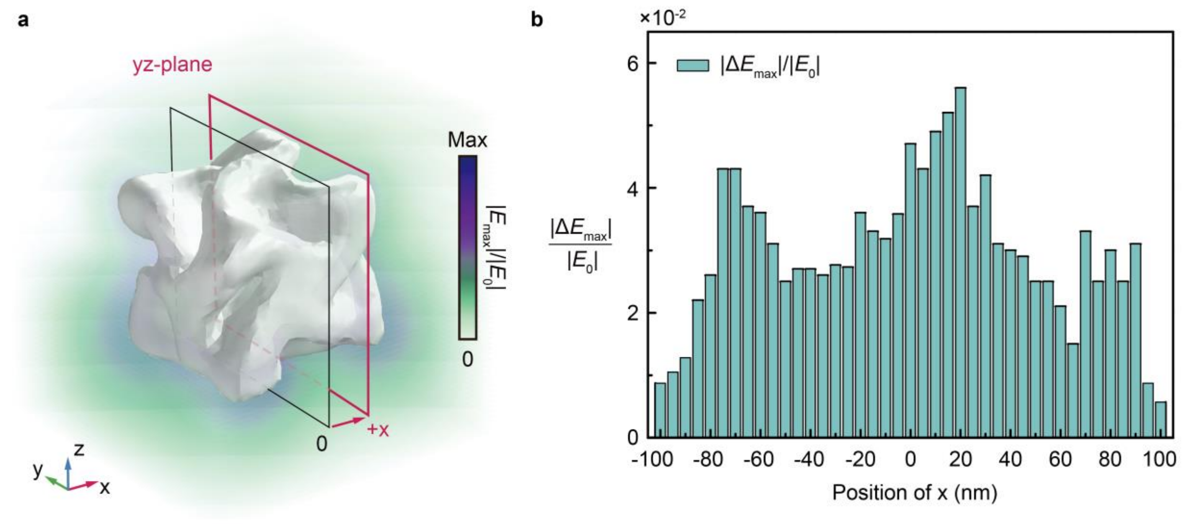

Supplementary Fig. 20 | Simulated near-field distribution of 432 helicoid III nanoparticle based on BCDI result. a, The scattered electric-field distribution normalized by incidence electric-field (the incidence of x-axis linearly polarized light at 630 nm) **b,** the difference of maximum scattered electric-fields of 432 helicoid III between the Drude-Sommerfeld (E_{DS}) and strain-induced model (E_{SI}) as the function of position x ($E_{max, DS}$ - $E_{max, SI}$ =ΔE_{max}).

Supplementary Table 1.

Parameters	Value
K	0.13
v_F	1.41×10^{6} m/s
$1/\gamma_0$	9.3×10^{-15} s
m_{eff}	$0.99\times m_{electron}$

Supplementary Table 1 | Parameters for the dielectric constant of the nanosized bulk gold.
