# Supplementary material for: Strain and crystallographic identification of the helically concaved gap surfaces of chiral nanoparticles
Source: Nat Commun. 2023 Jun 17;14:3615. doi: 10.1038/s41467-023-39255-1 (PMC10276881; doi:10.1038/s41467-023-39255-1)
Supplement: Supplementary file 2 — Description of Additional Supplementary Files Document [file 41467_2023_39255_MOESM2_ESM.pdf]

Description of Additional Supplementary Files Document

Supplementary Movie. 1 | Terrace area fitting procedure for cubic nanoparticle. Surface Miller-index of cubic nanoparticle with varying rotation angle along **+y′** (in Supplementary Fig. 4a) from -60° ~ 60° with 2° step size. At the center, the overall Miller-index of the plane becomes {100} series except for the edge and corner of the nanoparticle that produces the "most red" color-coding result.
